# Prioritization of Information Security Controls through Fuzzy AHP for Cloud Computing Networks and Wireless Sensor Networks

**DOI:** 10.3390/s20051310

**Published:** 2020-02-28

**Authors:** Muhammad Imran Tariq, Shakeel Ahmed, Nisar Ahmed Memon, Shahzadi Tayyaba, Muhammad Waseem Ashraf, Mohsin Nazir, Akhtar Hussain, Valentina Emilia Balas, Marius M. Balas

**Affiliations:** 1 Department of Computer Science and Information Technology, Superior University, Lahore 54000, Pakistan; 2 College of Computer Science and Information Technology (CCSIT), King Faisal University, Al-Ahsa 31982, Saudi Arabia; nmemon@kfu.edu.sa (N.A.M.); shakeel@kfu.edu.sa (S.A.); 3 Department of Computer Engineering, University of Lahore, Punjab 54000, Pakistan; shahzadi.tayyaba@dce.uol.edu.pk; 4 Department of Physics (Electronics), Government College, University of Lahore, Punjab 54000, Pakistan; 5 Department of Computer Science, Lahore College for Women University, Punjab 54000, Pakistan; mohsin.nazir@lcwu.edu.pk; 6 Department of Information Technology, Quaid-e-Awam University of Engineering, Science and Technology, Nawabshah 67480, Pakistan; jalbaniakhtar@quest.edu.pk; 7 Department of Automation and Applied Software; Aurel Vlaicu University of Arad, 310130 Arad, Romania; valentina.balas@uav.ro (V.E.B.); marius.balas@uav.ro (M.M.B.)

**Keywords:** wireless sensor networks, fuzzy logic, multi-criteria decision making, analytical hierarchy process, ISO/IEC 27002:2013, risk assessment, information security controls

## Abstract

With the advent of cloud computing and wireless sensor networks, the number of cyberattacks has rapidly increased. Therefore, the proportionate security of networks has become a challenge for organizations. Information security advisors of organizations face difficult and complex decisions in the evaluation and selection of information security controls that permit the defense of their resources and assets. Information security controls must be selected based on an appropriate level of security. However, their selection needs intensive investigation regarding vulnerabilities, risks, and threats prevailing in the organization as well as consideration of the implementation, mitigation, and budgetary constraints of the organization. The goal of this paper was to improve the information security control analysis method by proposing a formalized approach, i.e., fuzzy Analytical Hierarchy Process (AHP). This approach was used to prioritize and select the most relevant set of information security controls to satisfy the information security requirements of an organization. We argue that the prioritization of the information security controls using fuzzy AHP leads to an efficient and cost-effective assessment and evaluation of information security controls for an organization in order to select the most appropriate ones. The proposed formalized approach and prioritization processes are based on International Organization for Standardization and the International Electrotechnical Commission (ISO/IEC) 27001:2013. But in practice, organizations may apply this approach to any information security baseline manual.

## 1. Introduction

Recently, two evolving technologies, cloud computing and Wireless Sensor Networks (WSNs), have been integrated. Cloud computing is referred to as a pay-per-use model and has servers that are connected to the Internet. It is the core responsibility of the servers to respond to every request of the user at any time [1,2]. Furthermore, cloud networks permit access to its offered services via the Internet through any handheld device or fixed device located anywhere in the world. Wireless sensor networks have also performed a great role in the field of information technology by enabling cloud organizations to strengthen their monitoring systems. Wireless sensor networks provide uninterrupted and distributed operations that are important in the field of cloud computing and conventional networks. Both technologies are opposite and cover each other’s drawbacks. At present, much research has already been conducted regarding the integration of wireless sensor networks with cloud computing. Many organizations have also integrated both technologies based on their requirements [2,3,4,5,6,7,8]. It is paramount to add here that still no information security standard exists with information security controls for wireless sensor networks. Therefore, the majority of organizations apply conventional network information security standards on WSNs. Hence, this study considered the same Information Security Controls (ISCs) for wireless sensor networks that are deployed for information security in cloud computing networks for evaluation and prioritization. 

Information security is becoming more important day by day, and it is also the foundation for cloud networks integrated with the wireless sensor networks of an organization. The key aim of information security decision makers is to protect the cloud networks, WSNs, and the assets of the organization through information security risk assessment and the maintenance of the confidentiality, integrity, and availability of resources [9,10,11]. There are many risk assessment frameworks that depend upon qualitative methods and proper guidelines. Therefore, testing of ISCs on the basis of these risk assessment frameworks requires more time and costs. The ultimate goal of the information system in an organization is to improve all operations and simplify the decision process. Information Security Risk Management (ISRM) always played a critical role in the assessment of security risks associated with business operations. Also, it determined the selection of the most relevant information security controls [12,13]. In the past, information security experts had a habit of selecting all types of ISCs without considering the risks, attacks, costs, effectiveness, mitigation time, exploitation time, and maintenance time [14]. 

The risk assessment process has many steps such as identification of the ISCs to be tested, evaluation to check their efficiency, analysis of the test results, and improvement of the ISC on the basis of the test recommendations [15]. Several types of risk management methods and techniques have been developed to solve these issues by applying qualitative and quantitative modeling. Quantitative techniques that depend upon decision-making criteria are still lacking [16].

In the real world, every innovation has many obstacles. The challenge is to make the best decision among different alternatives and controls [17]. These alternatives are sometimes clashing in nature and increase the complexity of decision making [18]. To overcome all the limitations and choose a definitive solution to the problem is a really difficult task [19,20]. The difficulty in making decisions arises when the obstacles are not natural or are ambiguous, uncertain, and confusing. The proposed methods of this study can be used to solve actual problems that organizations face in real time with a series of restrictions that are classified into a Multi-Criteria Decision Making (MCDM) method [21,22]. 

The process of evaluating and selecting the most efficient information security controls for an integrated WSN–cloud network from existing information security standards can be seen as tricky and challenging. Baseline manuals provide very little guidance on how to select the best set of ISCs to provide required security against threats. Obviously, this set of ISCs should meet the security and privacy requirements of organizations. The selection of ISCs must be driven according to organizational requirements and the linked security needs. The information security policy of the organization must be composed of security requirements and a list of controls that have the capability to provide the required privacy and security protection. If a set of ISCs has been properly evaluated and testified to cater to an organization’s needs, then the trust needed for WSN–cloud networks can be created. Furthermore, during risk management processes, an organization needs to ensure that the best set of ISCs is identified, installed, and operated accurately. 

Under many contradictory obstacles, the decisions made by humans are not reliable, because the human brain is only capable of evaluating and acting on a limited amount of information at any given moment [17]. To help decision makers solve actual problems for organizations, Thomas Sati (1980) [23] introduced the Analytical Hierarchy Process (AHP). This approach is based on the comparison of pairs between an alternative and a best possible alternative. The strength of the AHP lies in its neutral and logical classification and its flexibility to integrate various functions such as the deployment of quality functions, linear programming, and fuzzy. The benefit of the AHP methodology in conjunction with fuzzy logic is called fuzzy AHP which is the most important method of the multi-criteria decision-making methodology for various types of applications [24]. The fuzzy AHP approach helps to make decisions with various inclinations, fuzziness, and vulnerability. Research has shown the fuzzy AHP philosophy and furthered the supreme utilization of it [25]. It is practical for dealing with uncertainty, complexity, and decision making for complex issues of a controversial nature [26]. 

The structure of the article is as follows: Section 2 is related to the integration of wireless sensor networks with cloud computing. Section 3 reviews previous approaches for the selection of ISCs in organizations. Section 4 presents the AHP, and Section 5 discusses fuzzy AHP along with the model. Section 6 covers the fuzzy AHP methodology, Section 7 elaborates on the application of fuzzy AHP for the selection of information security controls, Section 8 regards sensitivity analysis, and Section 9 compares the fuzzy AHP technique with other methodologies. Finally, Section 10 summarizes and concludes the paper with a suggestion for further research.

## 2. Integration of Wireless Sensor Networks with Cloud Computing

The goal of WSNs is to sense the environment in which it is installed and send data to the user. As the number of internet and cloud computing users is growing rapidly, there is a dire need to provide WSN services to this fast growing community [27]. 

Cloud computing is an adaptable, incredible, and cost-effective framework that provides real-time information to its clients however and whenever it is required, at varying levels of desired quality. The cloud is composed of software, infrastructure, platforms, storage, systems, and interfaces that empower the conveyance of computing services. Likewise, it is very easy to transfer the information received from wireless sensor nodes to the internet/cloud networks through Simple Object Access Protocol (SOAP), emails, short message service, and blogs, etc. [28]. By associating, assessing, and connecting these sensor systems, information can be concluded in real time, patterns can be anticipated, and unsafe circumstances can be avoided.

Wireless sensor networks (WSN) are a trendy domain of research due to the fact of their easy accessibility via the internet, any place and time. In WSNs, the sensors generate a large amount of data as input and appropriate action against the collected input is taken. Wireless sensor networks have generally been installed in various applications extending from smart industry, smart health, smart cities, smart environments, agriculture, habitat, indoor living, and greenhouses to climate and forest monitoring [29]. In all these fields, the sensors generate data in huge amounts that need some protocols to be collected, stored, and processed. The design of a WSN is structured in a specially appointed manner and is not adaptable to adjustment to different applications or situations, while the central issue is the equivalent remote observing utilizing sensor systems [30]. During the literature review, it was revealed that many researchers examined the way to connect the wireless sensor network to a cloud computing network [1,2,5,31,32,33,34,35]. In a WSN, the major issue with the deployment of sensors is the data storage, because sensors have temporary memory to store data and then send it to an end destination that can be a data repository [36]. The combination of a WSN with a cloud facilitates the network to store data on the cloud [37].

**Figure 1:**
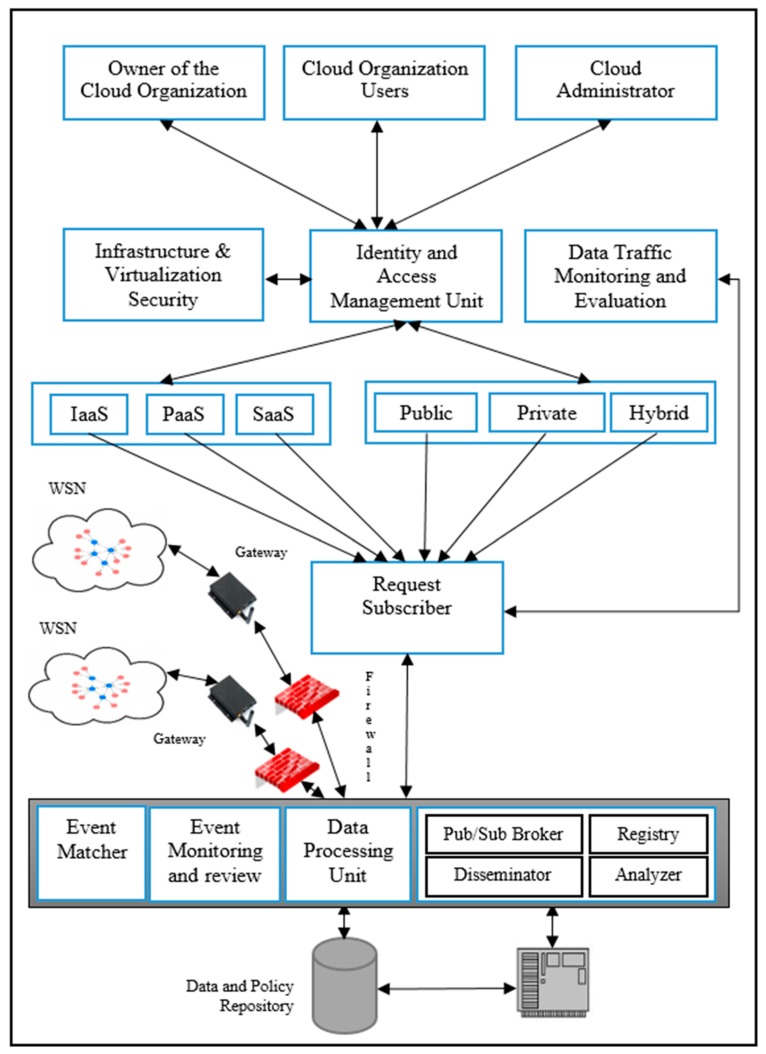
Integration of a WSN with a cloud network.

After an in-depth study of the literature, the authors of References [1,8,38] integrated a wireless sensor network with a cloud network. Their proposed framework has been updated, and the authors of this present article proposed their own WSN–cloud framework which is presented in Figure 1. 

The goals of the sensor–cloud integration framework are to assist the transfer of data/information received from a wireless sensor network to the cloud so that the events triggered by the sensors can be properly stored on cloud networks and to fully utilize the received data. The proposed integration of WSN–cloud framework components are an event matcher, data processing unit, event monitoring, review, publisher/subscriber broker, disseminator, registry, analyzer, request subscriber, identity and access management unit, policy repository, infrastructure and virtualization security, and data traffic monitoring and evaluation. In the first instance, all the users of the cloud will be connected to the sensor–cloud framework through a secured Identity and Access Management Unit (IAMU), and the users will be given access to the sensor–cloud network on the basis of the access policy stored in the IAMU against each account. After the successful granting permission, the users will be able to put their data access request; all such types of requests will be forwarded to the request subscriber component which will be further forwarded for subscription to the publisher and subscriber broker. 

The proposed integrated WSN–cloud framework components are event matcher, data processing unit, event monitoring, review, publisher/subscriber broker, disseminator, registry, analyzer, request subscriber, identity and access management unit, policy repository, infrastructure and virtualization security, and data traffic monitoring and evaluation.

### 2.1. Identity and Access Management Unit (IAMU)

**Figure 2:**
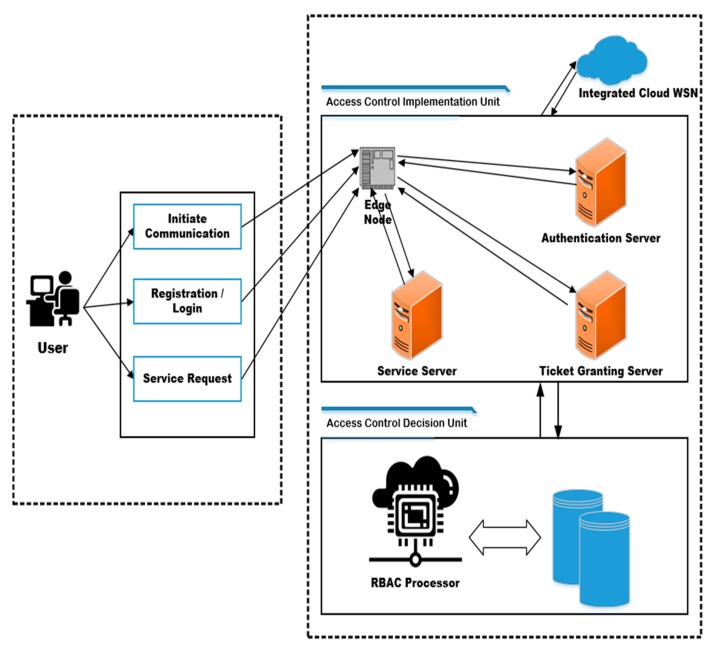
Overall identity and access management unit.

In the first instance, all the users of the cloud will connect to the sensor–cloud framework through a secured Identity and Access Management Unit (IAMU), and the users will give access to the sensor–cloud network on the basis of the access policy stored in the IAMU against each account. Figure 2 shows the overall IAMU system consisting of two major components that are the access control decision unit and the access control implementation unit. Many authors have already used the Kerberos authentication mechanism by introducing the Edge Node (EN) which uses the Diffie–Hellman (DH) public key algorithm. The DH is used to securely exchange cryptographic keys in the public network. 

The complete IAMU consists of Diffie–Hellman, Role-Based Access Control (RBAC), repository, service server, ticket granting server, authentication server, and Extensible Markup Language (XML). The IAMU has two primary purposes: the first is to ensure strong authentication between the user and cloud, and the second is to grant access to the cloud resources to the legitimate users using policy-based access [1]. 

The users will initiate their login/registration communication with the cloud through the Access Control Implementation Unit (ACIU) which will facilitate the user in the authentication process and access control over the cloud resources. 

#### 2.1.1. Access Control Implementation Unit (ACIU)

The ACIU is composed of edge node, authentication server, ticket granting server, and service server. All the user requests will be directly sent to edge node and then the authentication server. The Kerberos server will help the user to gain authentication via the authentication server. 

#### 2.1.2. Access Control Decision Unit (ACDU)

The access control decision unit is a very important unit of IAMU which primarily has RBAC. It enables users to obtain access to only those resources that are required for them to perform their job and also restrict users’ access to resources which do not pertain to them. The role of the user is already defined in the RBAC through the Java language, and the policies are defined in Extensible Markup Language (XML). The ACDU communicates with the ACIU through the service server [39]. 

### 2.2. Publisher/Subscriber Broker

After successfully being granting permission, users will be able to process their data access request, and all such types of requests will be forwarded to the request subscriber component which will further be forwarded for subscription to the publisher and subscriber broker. The framework is given in Figure 1. The publisher/subscriber broker is an independent unit and is not directly linked to the gateway. All the collected data from sensors are directly stored in the repository for further processing by the Data Processing Unit (DPU). The stored sensor data are properly formatted by the DPU, and only relevant data are extracted and then sent back to the repository. The index of each datum stored in the repository is assigned to the publisher/subscriber broker. The subscription requests of the user will be fulfilled through the request subscriber (RS) and event matcher. The goal of the event matcher is to handle mapping related issues between the RS and published data. After successful mapping, the publisher/subscriber broker will initiate the fetching process of data from the data repository, and the user may access the data through a cloud interface. 

### 2.3. Data Processing Unit (DPU)

The wireless sensor nodes generate data and send the data to the DPU through the General Packet Radio Service (GPRS) Gateway after filtration/security checking through the security firewall. The DPU processes the received data into a storage format and adds the data into the data and policy repository. The event monitoring and review component is responsible for continuously review and monitor of the quality of the received data and, in the case of any changes in the policy, review of the policy that is stored in the data and policy repository [38]. Whenever a new event is published by the publisher broker, the event matcher evaluates the event and starts the event matching process. When an event match becomes available to the user, further processing of the matched event is carried out if needed [1]. The function of the registry component is to store end-user subscriptions of the infrastructure, application, and platform service as well as the sensor data type. It also provides subscription details along with details of the applications to the disseminator component for the delivery of the event [34]. The publisher/subscriber component is mainly responsible for monitoring all events triggered through the WSN, processing each triggered event, and sending the events to the registered users through the cloud. The functioning of the analyzer component starts when the data or events are received by the publisher and subscriber broker. It determines to which cloud service model and deployment model the event belongs. It also provides information on whether the events are periodic or emergent. Thereafter, the analyzed events are passed to the disseminator to send appropriate events to the concerned users through the cloud service [34]. 

Data traffic monitoring and evaluation component of the framework keeps track of the utilization of the resources of cloud and request subscriber so that adequate resources can be distributed to a certain user. The function of the infrastructure and virtualization security component is to maintain privacy and security between the host operating system and guest operating systems as well as between wireless sensor network and cloud. 

### 2.4. Request Subscriber 

The purpose of the requesting subscriber is to deal with the requests of users. The RS module verifies the request of users regarding access to sensor data stored in the data repository. All service requests of the users are passed to the request subscriber module which after verification of the request, sends it to the publisher/subscriber for further mapping of the index data. 

### 2.5. Flow of Data Among Framework Components

**Figure 3:**
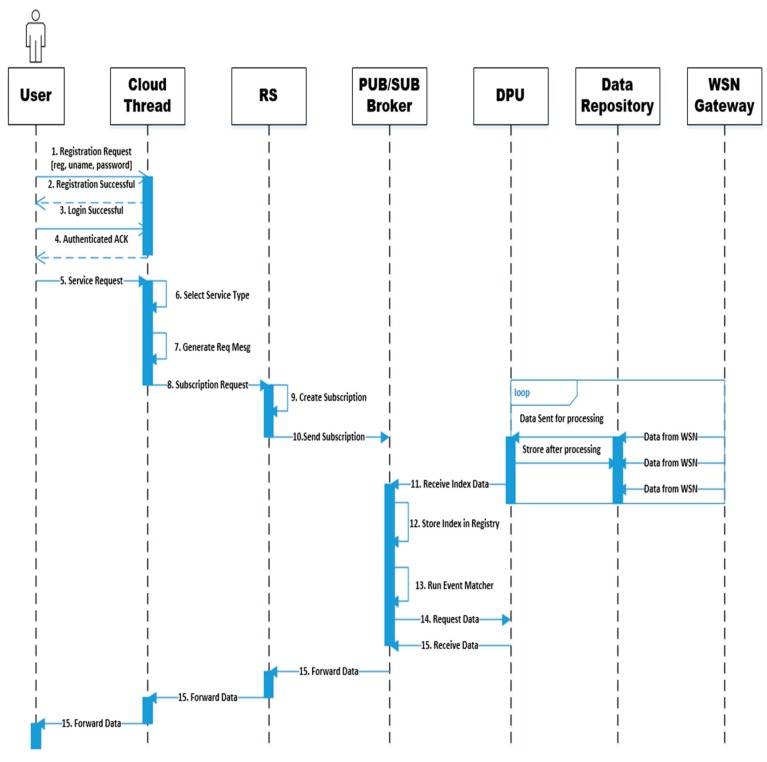
Sequence diagram of the proposed framework.

The sequence diagram of the proposed framework is presented in Figure 3. It shows the interaction among the main relevant components for the proposed framework. 

The steps of the sequence diagram are given as:

1. A new user arrives in the system and attempts to communicate with the cloud network of the organization. Its requests will go to the cloud threat wherein the edge node will first deal with the request. It is pertinent to add here that both the user and edge node will generate a common secret key by using the DF algorithm. The user sends its details (i.e., registration, username, and password) duly encrypted through a common secret key. The user’s registration details will be stored in the authentication server that is part of the ACIU.
2. Acknowledgment regarding successful registration of the user based upon information stored in the authentication server of ACIU will be sent to the edge node and then sent to the user. Thereafter, the user will generate a public/private key pair for the cloud. The ACIU decrypts the message and saves the public key in the authentication server against username. The encrypted message received from the cloud can be decrypted using the cloud’s public key.
3. The existing user will send a login request to the cloud thread wherein the edge node will deal with it. The user will provide encrypted usernames and passwords after establishing a connection through the Diffie–Hellman algorithm. User details will be going to the authentication server of the ACIU. User details will go to the authentication server of the ACIU.
4. Successful authentication Acknowledgement is sent to the user. Thereafter, the complete encryption process will be completed, as given above, to secure the communication between the user and the cloud.
5. After a successful login with the cloud network, the user will initiate a service request to the cloud network.
6. Thereafter, the cloud network will find the appropriate service type.
7. The cloud network will generate a corresponding request message.
8. After finding a suitable service type and generating a request message, the request of the user will be sent to the request subscriber.
9. The requesting subscriber will combine the request and create a new subscription on the basis of information received from the cloud network.
10. The subscription request will be sent to the publisher/subscriber broker.
11. The WSN gateway continuously sends sensor data to the data repository and the DPU quickly obtains sensor data for processing and then again stores the data in the data repository. The entire process is not time dependent in the proposed framework and can occur at any time. Therefore, a loop sign is used in the sequence diagram. The DPU will uninterruptedly send the prepared index of the data to the publisher/subscriber broker.
12. The index of the data will be stored in the registry of the publisher/subscriber broker.
13. Thereafter, the publisher and subscriber broker will initiate the event matcher to trace the same published data for received subscription requests.
14. If the publisher/subscriber broker finds relevant subscriptions related to the request, then the process of retrieving data from the DPU will be initiated.
15. The retrieved data from the DPU will be forwarded to the user through the request subscriber and the cloud network.

It is an entirely new idea to connect wireless sensor networks with cloud computing and, still, it is an area of research for organizations. It is impossible for organizations to connect each node of a WSN to the internet/cloud network.

The purpose of integrating a WSN with cloud is primarily to provide a storage service, recovery mechanism, and backup service to the sensed data/information. This can be achieved by sending all the sensed data and events to the cloud network through the internet. In this way, the user can access the sensed data from anywhere in the world, and the cloud network will also provide processing services to the sensed data for rapid and distributed processing. The integration of a WSN with a cloud also enables short-range hop networks to communicate with long-range networks and also enable WSNs to distribute their sensed data across the world. 

In the following, the authors of this article propose the architecture of a sensor–cloud framework and explain why we integrated a WSN with a cloud network, the purpose of the integration, and how the sensor–cloud framework works. 

## 3. Previous Approaches in the Selection of ISCs in Organizations

Based on Reference [40], previously, the process of identifying the most effective information security controls in an organizations has been a challenge, and many attempts have been made to formulate effective methods to meet this challenge. Risk Analysis and Management (RAM) is one such example. Risk analysis and management has widely been used as an effective approach to identify ISCs. Risk analysis and management conducts business analyses and risk assessments which help to identify information security requirements. Subsequently, RAM enlists the information security requirements and the proposed ISCs that can be implemented to mitigate the specific risks after conducting risk analysis and evaluation. However, RAM does not take specific constraints of the organization into consideration while selecting ISCs [41].

For example, when using RAM, organizations can identify 60 information security risks. However, it may be possible that management is unable to select and implement all the ISCs needed to address the identified risks due to the cost and budget constraints. Furthermore, there may not have enough resources within the organization to implement the ISCs. In this case, management must list all identified risks and determine the importance and severity level of each individual risk to the organization, taking into account cost/benefit analyses. The management of the organization must then explore new ways to determine/measure the relevance of these ISCs, taking into account the limitations.

Basic manuals or best practices are other approaches widely used by organizations to introduce minimum security controls in organizations. In Reference [42], best practice frameworks assist organizations in identifying appropriate ISCs. Furthermore, Reference [43] mentioned other best practice frameworks that contributed to the identification and selection of ISCs. 

The procedure of picking the most efficient ISCs from available best practice frameworks can be a challenge. According to Reference [41], best practice frameworks do not guide users toward the selection of effective ISCs. In addition, the available frameworks also do not consider the specific constraints of the organization such as cost, budget, effectiveness, suitability, scheduling, and resource limits. Other formal methods used in the past, such as ad hoc or randomized approaches, can lead to the inclusion of unnecessary controls and/or the exclusion of necessary controls.

The random approach is a less formalized method. The use of this method leads to the inclusion of unnecessary controls and the non-inclusion of necessary controls. Regarding the importance of selecting effective controls for information security, most organizations choose the most recent approaches and structures. For example, Reference [44] used the control information security checks. However, they used hypothetical variables to show a numerical example in their case study. Therefore, there is a lack of these information security checks.

The identification and selection of ISCs in light of the above clearly states that organizations are not able to protect the Confidentiality, Integrity and availability of their information [42]. In order to increase the effectiveness of the selection process and prioritization for ISCs, new methods are required to be developed that save time by considering the main factors (e.g., cost, budget, effeteness, and suitability) which undoubtedly influence the selection of ISCs.

In the literature review, it was clear that ISC selection is mainly based on costs, effectiveness, suitability, budget, and resource availability. In other words, ISC in organizations are selected by the administration when the benefits of its implementation are within budgetary limitation. Equally important, cost and budget can influence the possible selection of ISCs.

The implementation of ISCs can require specific methods, time, and costs. Finally, staff availability often determines whether ISCs can be selected or not. The effective implementation of the security in an organization may require the identification and adoption of the most appropriate and effective ISCs, taking into account the problems presented above.

The problems of evaluating and selecting information security controls require attention in practice. To address this problem, a number of approaches have been proposed by researchers using Multi Criteria Decision Making which deals with the selection of the best solutions amongst different alternatives/substitutes according to their attributes and objects. Multi-Objective Optimization by Ratio Analysis (MOORA) was introduced in Reference [45]. The Multi Criteria Decision Making (MCDM) presents different techniques such as the analytical hierarchy process (AHP). The AHP, developed in Reference [46], deals with how to define the relative significance of a set of activities. The AHP method consists of three principles: (1) developing the structure of the proposed model; (2) conducting a comparative analysis of the alternatives and criteria; and (3) preparing a synthesis of the priorities.

The Decision-Making Trial and Evaluation Laboratory (DEMATEL) method was developed to find the weights of each inter-dependence value against each criterion. Briefly speaking, it was developed in the years 1972 and 1976, and the Science and Human Affairs Program funded this project. The DEMATEL method is basically based upon the causes and effects of the criteria and converts causes and effects criteria into an intelligible model [47].

The Analytic Network Process (ANP) is a general form of AHP, and it is used in MCDM. It is a multi-criteria theory used to obtain relative priority scales of numbers from discrete decisions. The ANP does not make an assumption about the higher and lower levels of elements [48].

A Technique for Order Preference by Similarity to Ideal Solution (TOPSIS) was established in Reference [49]. The basic idea of this technique is that the nominated alternative must have the shortest distance from the ideal positive and negative solutions [50]. The TOPSIS method supposes that each criterion inclines towards a monotonically increasing or decreasing utility [51]. Therefore, it is the easiest way to describe ideal positive and negative solutions. The Euclidean distance approach is used to proximate the ideal solution. Therefore, the series of comparisons in light of relative distances are made to order the preference amongst the alternatives [50].

The Preference Ranking Organization Method for the Enrichment of Evaluations (PROMETHEE) is an outranking method and is used for different kinds of ISCs. It can be simply modified for use as a group judgment aid, for example, by introducing diverse weighting schemes [52]. Thus, PROMETHEE is clear and easier to use, even by decision makers (DMs) who are not familiar with Multi Attribute Decision Making (MADM).

Vlse kriterijumska optimizacija I kompromisno resenje (VIKOR) is a multi-criteria technique that generates optimized solutions, and it has preference over other MCDM approaches. The VIKOR was developed by Opricovic [53] in 1998, and in 2002 Opricovic and Tzeng [54] introduced criteria to deal with complex systems [55]. The VIKOR governs the ranking list, its solution, and intervals of weight stability. Similar to the other methods, this method has also been used to rank and select the best candidate amongst alternatives in light of conflicting criteria. The VIKOR introduced a ranking index technique for multi-criteria to measure the closeness of the ideal solution [54,55].

During the literature review, different methods were found to compare and prioritize the alternatives to select the best one; however, in this dissertation, different methodologies can be used such as fuzzy TOPSIS [56,57], fuzzy AHP [56], fuzzy ANP [58], and fuzzy VIKOR [59]. There are a number of studies that discuss ISC selection and mathematical methods that have been used to rank the ISCs as shown in Table 1. After comparing these techniques, fuzzy AHP was chosen as the most convenient, best, and easy to use method, as it facilitates prioritizing the alternatives based on the rating of the individual decision makers, supports a hierarchy process as well as a huge number of criteria, and deals with ambiguities. 

Furthermore, to acquire and manage the human assessment of ambiguity, vagueness, and subjectivity, the linguistic variables in fuzzy sets were integrated into the assessment and selection process of the ISCs. This widespread opted hybrid MCDM technique was applied to evaluate and select the information security control. The AHP approach was used which was based on the weights obtained with the entropy method in a fuzzy decision-making environment. The linguistic variables were converted into fuzzy values; to make the criteria more important, fuzzy values were utilized to calculate fuzzy entropy weights. To obtain a comprehensive performance evaluation of the ISCs problem, these weights were used in the fuzzy AHP approach. Thereafter, the total score of each control against each criterion was obtained for the selection of best control and to make a decision. The proposed method is more effective and easier to apply and provides the best decision-making quality.

## 4. Analytical Hierarchy Process (AHP)

Analytical hierarchy process is a technique to solve the complex, unstructured problems by breaking them down into small components, reorganizing these components into hierarchic positioning, synthesizing the final judgment to evaluate which alternative have the highest-ranking, and also to influence the output of the situation. It utilizes a hierarchical structure to extract, deteriorate, compose, and control the complexity of the decision including numerous attributes, and it utilizes judgment or the decision maker’s opinion to gauge the relative worth or commitment of these characteristics.

The AHP was developed by Thomas Saaty [23], and it is the most efficient method to make very complicated decisions, and it also helps the decision maker to decide the priorities and make the best decision among the alternatives [17]. In AHP, pairwise comparisons are developed to reduce complicated decisions, and synthetization of the results are also taken. Furthermore, the AHP also assists in taking subjective and objective types of the decision. In the AHP model, weights are given to the decision makers. During the decision-making process, the alternatives are compared with each other by assigning that weight. The AHP is currently used in many different areas such as computer programming, information investigation, and fuzzy set theory [90].

Furthermore, the AHP has a very helpful methodology to examine the reliability of evaluators/decision makers and, therefore, the biases of decision makers can be reduced through this process [91]. 

**Figure 4:**
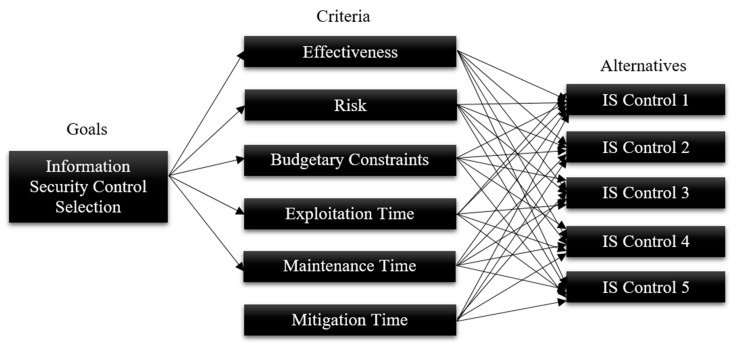
Hierarchy structure of the analytical hierarchy process (AHP).

Through the AHP, many assessment criteria and alternatives are discussed, and this helps in the selection of the best choice [17,92,93]. Figure 4 represents a hierarchy structure for the evaluation of ISCs utilizing the AHP method.

Figure 4 explains the processes of the AHP and all main criteria in a graphical format. In the AHP, the first process is to define the goals of the model which is the evaluation and best selection of the information security controls. After defining the goals, it is required to formulate the criteria of the evaluation. Here, in Figure 4, we defined six criteria in consultation with decision makers/experts. Every criterion was also assigned a certain weight which indicates its importance. The proposed criteria are applied to the alternatives to select the best candidate among all alternatives and also to prioritize the alternatives. Figure 4 shows five information security controls as alternatives. The best information security control and ranking of the information security controls must be done according to the six criteria.

The AHP gives weight to each criterion. Extra importance is given to the object that has a higher weight. Next, for a fixed paradigm, the AHP doles out a score to every alternative as indicated by the evaluator’s pairwise examinations of the choices dependent on that rule [13]. The better performance of the criteria is examined on the basis of the higher score with respect to each criterion. At last, the AHP combines the weights and the scores of the alternatives; in this manner, the score of each alternative is evaluated out of a total score it obtained against each criterion [25].

The primary goal of the AHP is to select an alternative that best satisfies a given set of criteria out of a set of alternatives or to determine the weights of criteria in any application. The AHP scales the weights of attributes at each level of the hierarchy with respect to a goal using the decision maker’s (experts’) experience and knowledge in a matrix of pairwise comparison of attributes. The usual application of AHP is to select the best alternative from a discrete set of alternatives. 

The main objective of the AHP is to choose an alternative that best fulfills a given arrangement of criteria out of many alternatives or to decide the weight of the criteria. The second objective of the AHP is to weights of sub-criteria at each degree of the hierarchy order regarding an objective utilizing the specialists’ experience. The AHP is the best methodology to select the best alternative from a set of different types of alternatives. 

## 5. Fuzzy Analytical Hierarchy Process (FAHP)

In the real world, there is ambiguous knowledge that is not natural. It is ambiguous, false, inexperienced, and often involves information that is unnecessary by nature. Humans give satisfactory possible answers that may not be definitive [92]. Zadeh [93] introduced the Fuzzy Set Theory (FST) to deal with uncertainty which used in decision-making processes by decision makers through linguistic terminology and degree of membership. It is an extension of classical work where the membership of the elements varies, and FST is based on logic zero false one truth which is sometimes inadequate to explain human reasoning. An important contribution of FST is its ability to represent ambiguous data. This diffuser set combines human reasoning with the use of data and uncertainty to make decisions. It was specifically designed to represent mathematics in order to represent uncertainty and ambiguity and to provide formal tools to solve many problems [94].

The Fuzzy approach of the AHP may be utilized to overwhelm the defect of the AHP, for example, by reducing accuracy because of ambiguous human ideas that are not handled [12]. In the AHP method, two alternatives are matched with each other and a numerical value is allocated to designate the degree of relevance between them [93,94]. In the fuzzy analytical hierarchy process, priority is characterized by intervals which cross each other. This improves the precision of the final decision, keeping human nature unsuitable for health [95,96,97].

## 6. Fuzzy AHP Methodology

**Figure 5:**
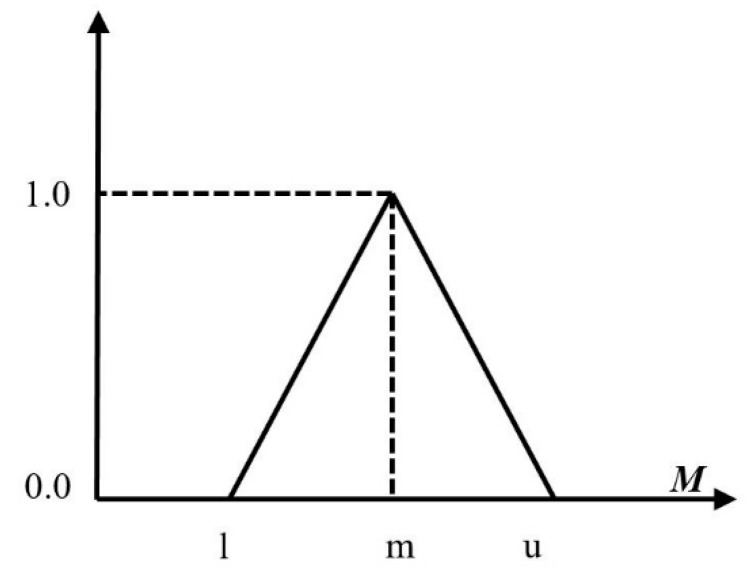
Triangular fuzzy number.

In the FAHP, alternatives are evaluated by using triangular fuzzy (TFN) and for the prioritization of alternatives and then to cope with the problems of the AHP. It was extended and a synthetic pairwise evaluation function was introduced [98]. The triangular fuzzy number denoted with M is shown in Figure 5.

Most of the researchers and academicians represented triangular fuzzy numbers by *M* = *(l, m, u)*. The *l* is used to express the smallest possible value, *m* is used to express the closest value, and *u* is accordingly used to express the largest possible value. The membership function is described as:

(1) $$\mu \;\left(\bar{M}\right)=\{\begin{array}{c} 0,\;x<l\\ \left(x-l\right)/\left(m-l\right),\;l\leq x\leq m\\ \left(u-x\right)/\left(u-m\right),\;m\leq x\leq u\\ 0,\;x>u \end{array}$$

There are many operations that can be performed on triangular fuzzy numbers, but the main operations that have been used in this study are illustrated below. Let us assume two triangular fuzzy numbers *M_1_* = *(l_1_, m_1_, u_1_)* and *M_2_* = *(l_2_, m_2_, u_2_)* then:

(2) $$\left(l_{1},\;m_{1},u_{1}\right)+\left(l_{2},\;m_{2},\;u_{2}\right)=\left(l_{1}+,\;l_{2},\;m_{1}+m_{2},\;U_{1}+U_{2}\right)$$

(3) $$\left(l_{1},\;m_{1},u_{1}\right)*\left(l_{2},\;m_{2},\;u_{2}\right)=\left(l_{1}*l_{2},\;m_{1}*m_{2},\;U_{1}*U_{2}\right)$$

(4) $${\left(l_{1},\;m_{1},u_{1}\right)}^{-1}=\left(\frac{1}{U_{1}},\frac{1}{m_{1}},\frac{1}{l_{1}}\right)$$

Suppose $X=\left\{\;x_{1},x_{2},\;x_{3}\ldots \ldots \ldots ..x_{n}\right\}$ is an object set and the goal setting is supposed as $G=\left\{g_{1},g_{2},\;g_{3}\ldots \ldots \ldots ..g_{n}\right\}$. Next, according to Chang’s [19] method, each object is taken from the list of the objects and an extended analysis of each goal is performed on each object. After the implementation of the extent analysis, the m extent analysis value for each object can be obtained using the following triangular fuzzy numbers:

$$M_{gi}^{1},\;M_{gi}^{2},\;\ldots \ldots ..\;M_{gi}^{m},\;i=1,2,3\ldots .,\;n.$$

We may represent the fuzzy synthetic extent value in respect of *i*th object as:

(5) $$S_{i}=\;\overset{m}{\underset{j=1}{\sum }}M_{gi}^{j}⊕\;{\left[\overset{n}{\underset{i=1}{\sum }}\overset{m}{\underset{j=1}{\sum }}M_{gi}^{j}\right]}^{-1}$$

To get $\sum_{j=1}^{m}M_{gi}^{j}$, a fuzzy addition operation is required to be performed on *m* above the extent analysis values for a specific matrix using Equation (6):

(6) $$\overset{m}{\underset{j=1}{\sum }}M_{gi}^{j}=\left(\overset{n}{\underset{j=1}{\sum }}l_{j},\;\overset{n}{\underset{j=1}{\sum }}m_{j},\;\overset{n}{\underset{j=1}{\sum }}u_{i}\right)$$

Furthermore, in order to get the ${\left[\sum_{i=1}^{n}X_{i}\sum_{j=1}^{m}M_{gi}^{j}\right]}^{-1}$, first, the fuzzy addition operation must be performed by considering Equation (7), and then the inverse of the vector on the basis of Equation (8) must be taken.

(7) $$\overset{n}{\underset{i=1}{\sum }}\overset{m}{\underset{j=1}{\sum }}M_{gi}^{j}=\;\left(\overset{n}{\underset{j=1}{\sum }}l_{j},\;\overset{n}{\underset{j=1}{\sum }}m_{j},\;\overset{n}{\underset{j=1}{\sum }}u_{i}\right)$$

(8) $${\left[\overset{n}{\underset{i=1}{\sum }}\overset{m}{\underset{j=1}{\sum }}M_{gi}^{j}\right]}^{-1}=\;\left(\frac{1}{\sum_{j=1}^{n}u_{i}}\frac{1}{\sum_{j=1}^{n}m_{i}}\frac{1}{\sum_{j=1}^{n}l_{i}}\right)$$

After the determination of the synthetic extent, the next step is to determine the degree of possibility in the case of $M_{2}=\left(l_{2},m_{2},\;u_{2}\right)\geq M_{1}=\left(l_{1},\;m_{1},\;u_{1}\right)$. It can be calculated as follows:

(9) $$V\left(M_{2}\geq M_{1}\right)=\underset{y\geq x}{\sup}[\min\left(\mu_{M_{1}}\;\left(x\right),\;\mu_{M_{2}}\left(y\right)\right]$$

(10) $$V\left(M_{2}\;\geq M_{1}\right)=hgt\;\left(M_{1}⋂M_{2}\right)=\mu_{M2}\left(d\right)$$

The ordinate of the highest intersection point is denoted as d and is lying between $\mu_{M1}$ and $\mu_{M2}$.

(11) $$V\left(M_{2}\;\geq M_{1}\right)=hgt\;\left(M_{1}⋂M_{2}\right)=(l_{1}-u_{2})/\;\left(m_{2}-u\_2\right)-\left(m_{1}-l_{1}\right)\;$$

The term hgt is the height of fuzzy numbers on the intersection of $M_{1}$ and $M_{2}$.

**Figure 6:**
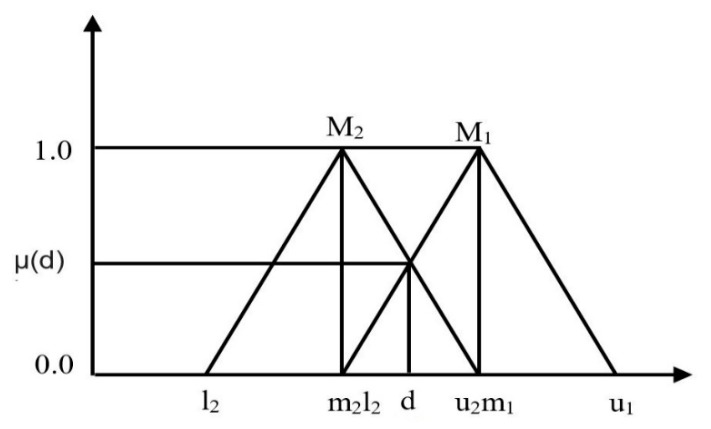
The intersection between M_1_ and M_2._

Figure 6 shows that the intersection of both triangular fuzzy numbers is denoted as (d) as ordinate, and it is the highest intersection point *D* between two fuzzy membership functions. 

To compute the smallest degree of possibility $M_{2}\geq M_{1}$, all fuzzy values $M_{i}=\left(1,2,\ldots .,\;k\right)$ are required to be compared using the following equation:

(12) $$V\;\left(M\geq M_{1},M_{2},\ldots .,\;M_{k}\right)=minV\left(M\geq M_{i}\right),\;\left(i=1,2,\ldots \ldots ,k\right)$$

Let us suppose, $d^{\prime }\left(A_{i}\right)=minV\;\left(S_{i}\geq S_{k}\right)$; for K=1,2,….,n, the weights can be calculated under:

(13) $$W^{\prime }=(d^{\prime }\left(A_{1}\right),\;d^{\prime }\left(A_{2}\right),\;\ldots \ldots ,\;d^{\prime }\left(A_{n}\right)\;\mathrm{where}\;A_{i}=\left(i=1,2,\ldots ..,n\right)\;\mathrm{and}\;n\;\mathrm{are}\;\mathrm{elements}$$

The next step is to normalize the vector, W′. The final priority weights vector of each alternative are calculated as follows:

(14) $$W={\left(d\left(A_{i}\right),\;d\left(A_{2}\right),\ldots \ldots \ldots \ldots d\left(A_{n}\right)\right)}^{T}\mathrm{where}\;W\;\mathrm{is}\;\mathrm{not}\;a\;\mathrm{fuzzy}\;\mathrm{number}.$$

The consistency index (CI) is the next step, and the following equation is used to calculate it.

(15) $$CI=\frac{\;\lambda_{max}-n\;}{n-1}$$

Finally, the consistency ratio (CR) is calculated as follows:

(16) $$CR=\frac{CI}{RI}$$

Here RI means Random Index table which is available on the Internet.

**Figure 7:**
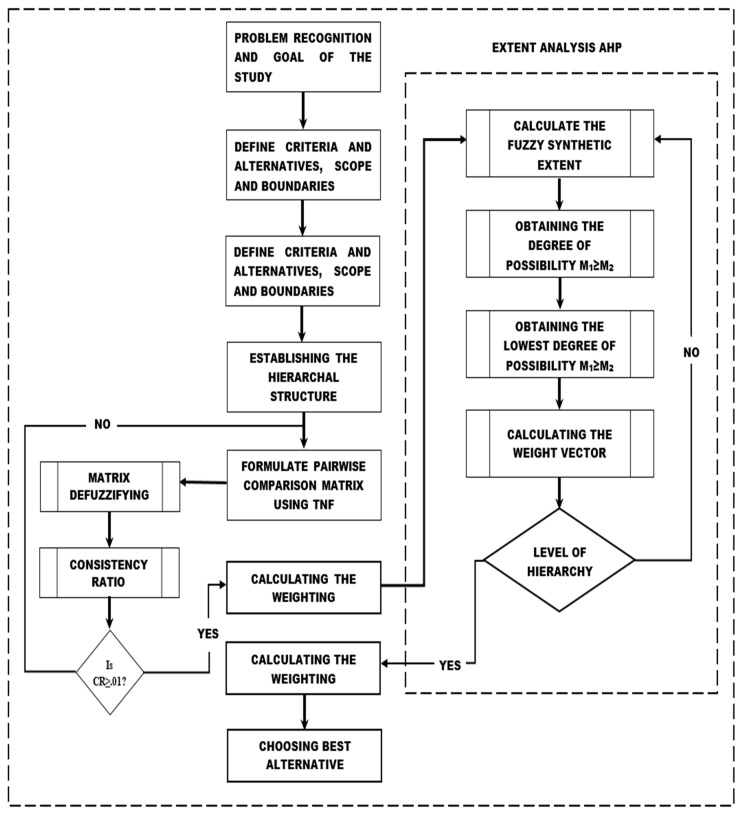
Fuzzy AHP methodology.

The specific steps of the FAHP and the step-by-step procedure adopted in this study to calculate the weights of the criteria are shown in Figure 7.

## 7. Application of the Fuzzy AHP for the Selection of Information Security Controls

**Figure 8:**
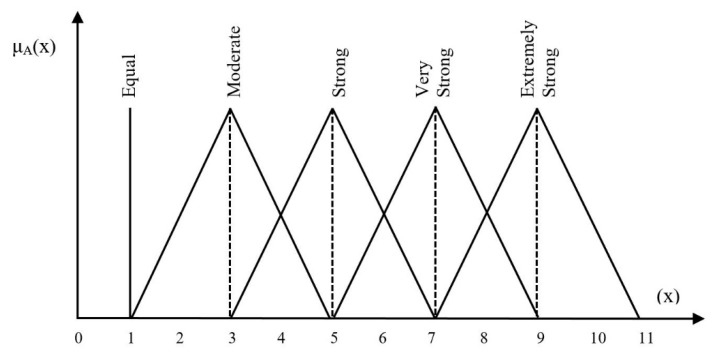
Linguistic scale triangular numbers for the relative importance.

An organization having a WSN–cloud-integrated network needs evaluation and prioritization of the information security controls to effectively implement security and privacy. In this situation with inadequate resources, the organization wants to use as much effort as possible to evaluate ISCs which are very important for risk management. The best selection of information security controls is a serious and very important task for organizations. The objective of this section is to choose the best ISCs among alternatives. The organization desires to take all essential possible criteria which are vital in the process of security control selection. For ISC selection and evaluation, there are seven main criteria determined by the decision makers’ team: implementation time (C1), effectiveness (C2), risk (C3), budgetary constraints (C4), exploitation time (C5), maintenance cost (C6), and mitigation time (C7). Secondly, we assigned them the weights of the criteria. The information security control that has the highest priority weight will be selected as the best information security control. The problem hierarchy determines how every alternative (information security control) is assessed through the predefined criteria. The preferences are determined using the triangular fuzzy scale (0 to 11). In this way, there is a very small chance of mistake and error as compared to the AHP. Figure 8 shows the fuzzy AHP model used in this study.

The linguistic scale was prepared after an in-depth literature review to develop a pairwise comparison matrix, as shown in Table 2.

In the next step, the values of the linguistic scale were transformed into TFNs as shown in Table 3. A pairwise comparison matrix was required to be developed in light of the opinions/preferences of the decision makers against each criterion. 

After the formalization of the above fuzzy pairwise comparison matrix, the next step was to calculate the weights of all the criteria with the support of the FAHP. Therefore, keeping in view the FAHP method, the synthesis values of the criteria were required to be calculated based on Equation (5).

S_C1_ = (6.33, 6.33, 6.33) * (0.010, 0.014, 0.022) = (0.063, 0.090, 0.142)

S_C2_ = (11, 21, 31) * (0.010, 0.014, 0.022) = (0.109, 0.3, 0.695)

S_C3_ = (6.20, 14.33, 23) * (0.010, 0.014, 0.022) = (0.061, 0.205, 0.505)

S_C4_ = (7.40, 11.67, 17) * (0.010, 0.014, 0.022) = (0.073, 0.167, 0.381)

S_C5_ = (5.40, 7.67, 11) * (0.010, 0.014, 0.022) = (0.053, 0.109, 0.247)

S_C6_ = (3.69, 4.07, 5.67) * (0.010, 0.014, 0.022) = (0.036, 0.058, 0.127)

S_C7_ = (4.60, 5, 7) * (0.010, 0.014, 0.022) = (0.046, 0.071, 0.157)

The next step is to calculate the degree of possibility by using Equation (10).

*V* (S_C1_≥S_C2_) = 0.136, *V* (S_C1_≥S_C3_) = 0.414, *V* (S_C1_≥S_C4_) = 0.474, *V* (S_C1_≥S_C5_) = 0.823, *V* (S_C1_≥S_C6_) = 1,

*V* (S_C1_≥S_C7_) = 1

*V* (S_C2_≥S_C1_) = 1, *V* (S_C2_≥S_C3_) = 1, *V* (S_C2_≥S_C4_) = 1, *V* (S_C2_≥S_C5_) = 1, *V* (S_C2_≥S_C6_) = 1, *V* (S_C2_≥S_C7_) = 1

*V* (S_C3_≥S_C1_) = 1, *V* (S_C3_≥S_C2_) = 0.810, *V* (S_C3_≥S_C4_) = 1, *V* (S_C3_≥S_C5_) = 1, *V* (S_C3_≥S_C6_) = 1, *V* (S_C3_≥S_C7_) = 1

*V* (S_C4_≥S_C1_) = 1, *V* (S_C4_≥S_C2_) = 0.671, *V* (S_C4_≥S_C3_) = 0.894, *V* (S_C4_≥S_C5_) = 1, *V* (S_C4_≥S_C6_) = 1,

*V* (S_C4_≥S_C7_) = 1

*V* (S_C5_≥S_C1_) = 1, *V* (S_C5_≥S_C2_) = 0.420, *V* (S_C5_≥S_C3_) = 0.661, *V* (S_C5_≥S_C4_) = 0.752, *V* (S_C5_≥S_C6_) = 1,

*V* (S_C5_≥S_C7_) = 1

*V* (S_C6_≥S_C1_) = 0.665, *V* (S_C6_≥S_C2_) = 0.070, *V* (S_C6_≥S_C3_) = 0.309, *V* (S_C6_≥S_C4_) = 0.331, *V* (S_C6_≥S_C5_) = 0.589,

*V* (S_C6_≥S_C7_) = 0.859

*V* (S_C7_≥S_C1_) = 0.832, *V* (S_C7_≥S_C2_) = 0.174, *V* (S_C7_≥S_C3_) = 0.418, *V* (S_C7_≥S_C4_) = 0.468, *V* (S_C7_≥S_C5_) = 0.731,

*V* (S_C7_≥S_C6_) = 1.

Using Equation (12), priority weights were calculated.

$d^{\prime }\left(C_{1}\right)=\min($0.136, 0.414, 0.474, 0.823, 1, 1) = 0.136

$d^{\prime }\left(C_{2}\right)=\min(1$, 1, 1, 1, 1, 1) = 1

$d^{\prime }\left(C_{3}\right)=\min(1$, 0.810, 1, 1, 1, 1) = 0.810

$d^{\prime }\left(C_{4}\right)=\min(1$, 0.671, 0.894, 0.1, 1, 1) = 0.671

$d^{\prime }\left(C_{5}\right)=\min(1$, 0.420, 0.661, 0.752, 1, 1) = 0.420

$d^{\prime }\left(C_{6}\right)=\min($0.665, 0.070, 0.309, 0.331, 0.589, 0.859) = 0.070

$d^{\prime }\left(C_{7}\right)=\min($0.832, 0.174, 0.418, 0.468, 0.731, 1) = 0.174

The priority weights were $W^{\prime }=\left(0.316,\;1,\;0.810,\;0.671,\;0.420,\;0.070,\;0.174\right)$, and the next step was normalizing the priority weights by using Equation (13) and W=(0.042, 0.304, 0.247, 0.205, 0.128, 0.021, 0.053) using Equation (14). The final weights of each criteria are given in Table 4.

The value of $\lambda_{max}=7.702$, and the value of the CI was calculated using Equation (15), and it was 0.117. Similarly, the value of CR was calculated using Equation (16), and it was 0.089 which is less than 0.1. 

### Evaluation of Alternatives with Respect to Each Criterion

The next step was to evaluate the alternatives. The ISO/IEC 27002:2013 has 114 information security controls, and it is not possible to demonstrate the detailed steps of 114 information security controls. Therefore, the authors decided to pick one domain of information security controls as alternatives and demonstrate a step-by-step calculation so that other information security controls could be evaluated using the same pattern. To accomplish this, we used the fuzzy AHP geometric means approach for synthetic pairwise comparison matrix as suggested by Buckley [99].

Step 1: Similar to the evaluation of the main criteria, the first step is to develop a pairwise comparison matrix, keeping in view the first criterion using Equation (17).

(17) $$A_{k}=\;\left[\begin{array}{ccc} d_{11}^{k} & d_{12}^{k}\ldots & d_{1n}^{k}\\ d_{21}^{k} & \ldots \ldots & d_{2n}^{k}\\ d_{n1}^{k} & d_{n2}^{k}\ldots & d_{nn}^{k} \end{array}\right]$$

Here, $d_{ij}^{k}$ demonstrates the liking of i criterion over j, by the k evaluators. 

The pairwise comparison matrix for implementation time criteria is given in Table 5.

Step 2: The next step was to calculate the fuzzy geometric means of the pairwise comparison matrix using the Equation (18).

(18) $$r_{i}={\left(\prod_{j=1}^{n}d_{ij}\right)}^{\frac{1}{n}},\;\;\;\;\;\;\;\;i=1,2,\ldots .n$$

$r_{i}={\left(\prod_{j=1}^{n}d_{ij}\right)}^{\frac{1}{n}}=\;\left[{\left(1*1*3*5\right)}^{\frac{1}{4}};\;{\left(1*3*5*7\right)}^{\frac{1}{4}};\;{\left(1*5*7*9\right)}^{\frac{1}{4}}\;\right]$

ri=(1.97, 3.20, 4.21)

Similarly, geometric means values of the fuzzy comparison matrix were calculated for the remaining alternatives. Table 6 shows the complete geometric means of Table 5. In the last 03 lines of the given table, a total of the alternatives is also given. Their reverse values are also shown and, according to the requirements of fuzzy triangular numbers, the order of the numbers should be sequential. 

Step 3: The next step is to calculate the fuzzy weights, $w_{i}$ of each alternative by using Equation (19).

(19) $$W_{i}=r_{i}*{\left(r_{1}*r_{2}*\ldots \ldots *r_{n}\right)}^{-1}=\left(lw_{i},\;mw_{i},\;uw_{i}\right)$$

The following steps are required to be performed first before calculating $w_{i}$. First, calculate the summation of each $r_{i}$ calculated in Table 6 under the “Total” heading; then, calculate the (-1) power of the total $r_{i}$ value as shown in the “Reverse” heading of Table 6; finally, calculate $w_{i}$ by multiplying each $r_{i}$ with the reverse values. $w_{i}=\;\left[\left(1.97*0.12\right);\;\left(3.20*0.18\right);\;\left(4.21*0.29\right)\right]=\;\left[0.237;0.564;1.205\right]$.

The fuzzy weights $w_{i}$ of each alternative in respect to the implementation time criteria are presented in Table 7.

Step 4: In the fourth step, the relative weights of the non-fuzzy numbers of the alternatives were calculated using Equation (20).

(20) $$M_{i}=\frac{lw_{i}+mw_{i}+uw_{i}}{3}$$

Step 5: The fifth step was to normalize the non-fuzzy numbers using Equation (21).

(21) $$N_{i}=\frac{M_{i}}{\sum_{i=1}^{n}M_{i}}$$

The results of the $M_{i}\;\mathrm{and}\;N_{i}$ are shown in Table 8. 

Step 6: The next step was to check the consistency index and the fuzzy pairwise comparison matrix. The value of $\lambda_{max}=4.4135$ and the value of the C) was calculated using Equation (15), and it was 0.045. Similarly, the value of CR = 0.050, calculated using Equation (16), which is less than 0.10. 

Step: 7: Here, we repeated steps 1 to 6 for the evaluation of each remaining alternative by using the equations given above. 

The pairwise comparison matrix for the effectiveness (C_2_) criteria is given in Table 9.

The results of the $r_{i},\;W_{i},\;M_{i}\;\mathrm{and}\;N_{i}$ is calculated in respect to C_2_ and presented in Table 10.

The CI = 0.046 and CR = 0.051.

The pairwise comparison matrix for the risk (C_3_) criteria is given in Table 11.

The results of the $r_{i},\;W_{i},\;M_{i\;}\mathrm{and}\;N_{i}$ was calculated in respect to the risk (C_3_) and is presented in Table 12.

The CI = 0.055 and CR = 0.061.

The pairwise comparison matrix for the budgetary constraints (C_4_) criteria is given in Table 13.

The results of the $r_{i},\;W_{i},\;M_{i}\;\mathrm{and}\;N_{i}$ was calculated in respect to the budgetary constraints (C_4_) and is presented Table 14. 

The CI = 0.073 and CR = 0.082.

The pairwise comparison matrix for the exploitation time (C_5_) criteria is given in Table 15. 

The results of the $r_{i},\;W_{i},\;M_{i}\;\mathrm{and}\;N_{i}$ is calculated in respect to the exploitation time (C_5_) and is presented in Table 16.

The CI = 0.081 and CR = 0.090.

The pairwise comparison matrix for the maintenance cost (C_6_) criteria is given in Table 17.

The results of the $r_{i},\;W_{i},\;M_{i}\;\mathrm{and}\;N_{i}$ is calculated in respect to the maintenance cost (C_6_) and presented in Table 18.

The CI = 0.035 and CR = 0.039.

The pairwise comparison matrix for the mitigation time (C_7_) criteria is given in Table 19.

The results of the $r_{i},\;W_{i},\;M_{i}\;\mathrm{and}\;N_{i}$ is calculated in respect to the mitigation time (C_7_) and is presented in Table 20. 

The CI = 0.012 and CR = 0.013.

**Figure 9:**
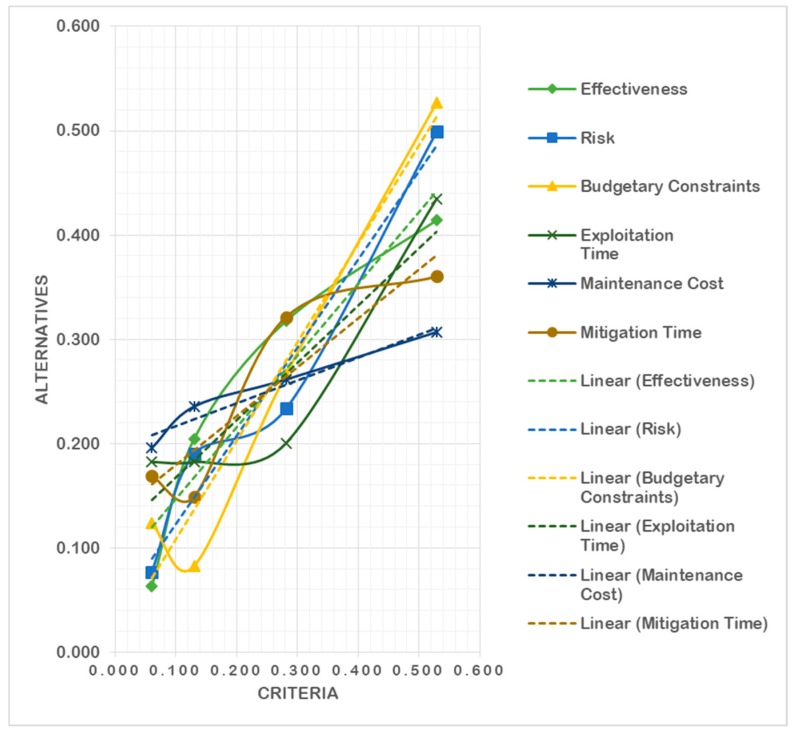
Final criteria weights in respect to all alternatives.

Step 8: This is the last step to calculate the final weights of all the alternatives by multiplying the matrix of the normalized weights of each alternative with the matrix of the criteria weights while evaluating in respect to each criterion. The final weights of each alternative are calculated and shown in Table 21 and depicted in Figure 9.

The complete steps for the weighting of criterion through the fuzzy analytical hierarchy process using the extended analysis method and prioritization of the alternatives/information security controls were conducted. The FAHP using geometric means was used. The details are given above and in light of these steps, all the information security controls of ISO / IEC 27002:2013 were evaluated and prioritized. The results are shown in Table 22.

The above results show that the controls have different ranks but fall in the same preference band. This is because similar preference band controls had almost the same results. The information security controls of a higher preference band must be given higher priority while implementing these controls, etc. 

## 8. Sensitivity Analysis

Sensitivity analysis is a widely adopted tool. It is mostly used in financial modeling to understand how various types of independent values affect dependent values under specific conditions. It covers a range of fields such as economics, social science, engineering, and geography. A sensitivity analysis is also known as what–if analysis, and its main goal is to know the uncertainty in the output by varying the values of the input. In multi-criteria decision-making methodology, each criterion has an assigned weight, and after getting final results/ranking, it is essential to check whether a slight variation in the weight of the criteria has a big impact on the output. If a slight change occurs in the output, then there is no issue. But if a large change occurs on the output due to the fact of minor change, then it creates an issue in the model.

During the sensitivity analysis, the weights of the seven criteria were analyzed individually by increasing the weight of the criteria from 1% to 5%. In this analysis, weights up to 10% would be increased to see the impact on the results. It is pertinent to mention here that we selected 7 information security controls from the 16.1 domain of the ISO/IEC 27001:2002 as a sample to perform the sensitivity analysis weight varying technique.

### 8.1. Implementation Time

**Figure 10:**
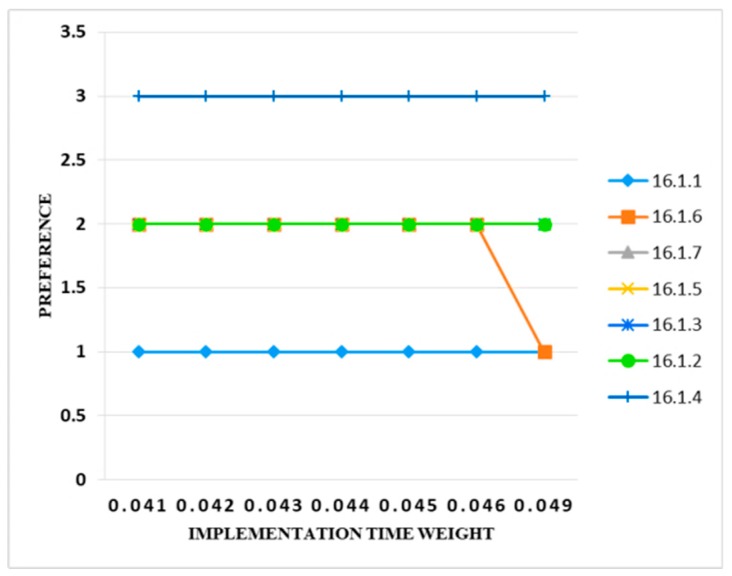
Sensitivity analysis of preference and implementation time weight.

The initial value of the implementation time criteria was 0.04. The weight of the said criteria was increased to 0.041, then it was observed that there was no change in the output. Similarly, the weight of the criteria increased up to 0.048 and no change in the output was found. This shows that there was no uncertainty in the model. The preference band assigned to the was fine. When the weight of the implementation time criteria increased to 10%, then it was observed that the preference band of only information security control (i.e., 16.1.10 were changed from preference band 2 to 1. Figure 10 illustrates these results.

### 8.2. Effectiveness 

**Figure 11:**
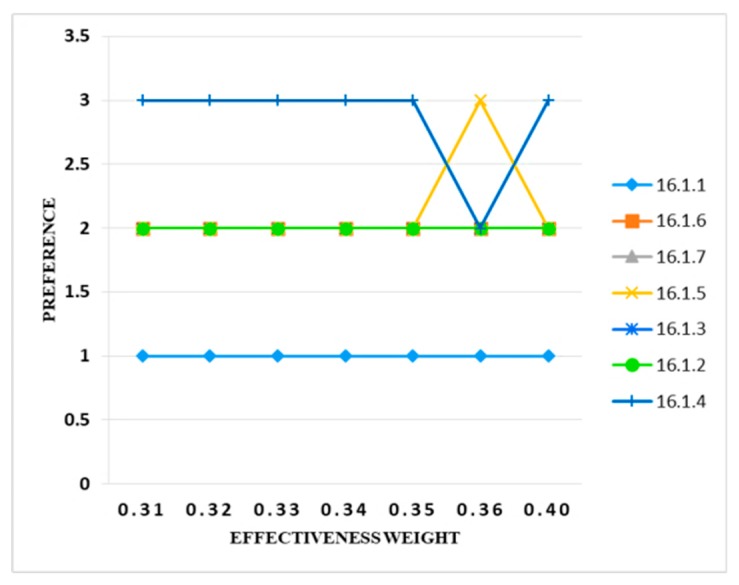
Sensitivity analysis of preference and effectiveness weight.

The initial value of the effectiveness criteria was 0.31. The weight of the said criteria increased to 0.32, then it was observed that there was no change in the output. Similarly, the weight of the criteria increased to 0.35 and no change in the output was found. When the weight of the criteria was set to 0.36, the preference band of the two information security controls (i.e., 16.1.5 and 16.1.4) were changed. This shows that there was no uncertainty in the model. The preference band assigned to the criteria was fine. When the weight of the implementation time criteria increased to 10% (0.40), it is observed that the preference band of the same two variables changed from preference band 3 to 2 and 2 to 3, respectively. Figure 11 illustrates these results.

### 8.3. Risk

**Figure 12:**
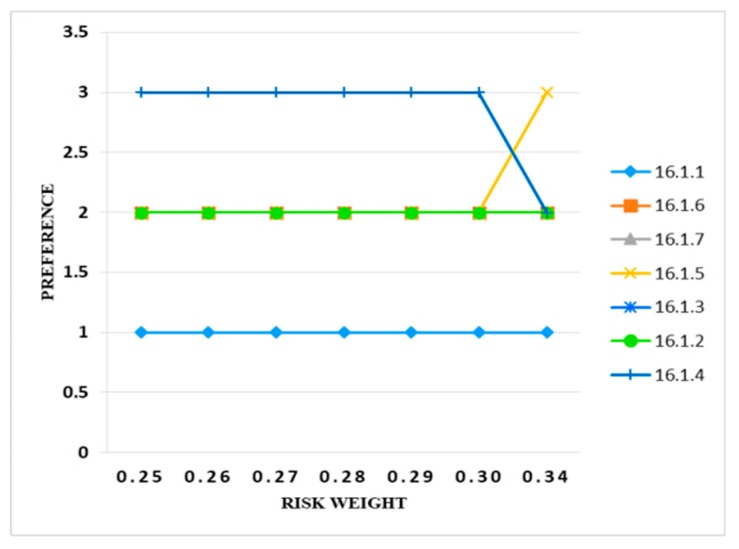
Sensitivity analysis of preference and risk weight.

In order to analyze the results, the weight was required to be increased by 1% and up to 10%. There was no change in the weights from 0.25 to 0.30, whereas the preference band of the only two controls (i.e., 16.1.4 and 16.1.5) was changed. The results depicted in Figure 12 shows that there was no change in the preference band of the information security controls if the risk criteria varied from 1% to 10%. 

### 8.4. Budgetary Constraints

**Figure 13:**
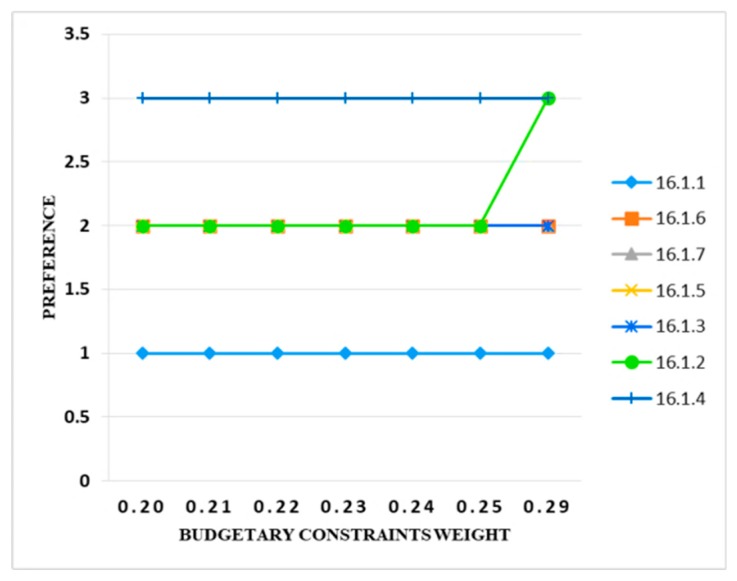
Sensitivity analysis of preference and budgetary constraints weight.

When the weight of the budgetary constraints increased from 1% to 10%, it was observed that only one information security control (i.e., 16.1.2) changed its preference band from 2 to 3 with a weight increase of up to 10%. It clearly shows that the criteria were stable and that there was no uncertainty in the model. Figure 13 illustrates these results. 

### 8.5. Exploitation Time

**Figure 14:**
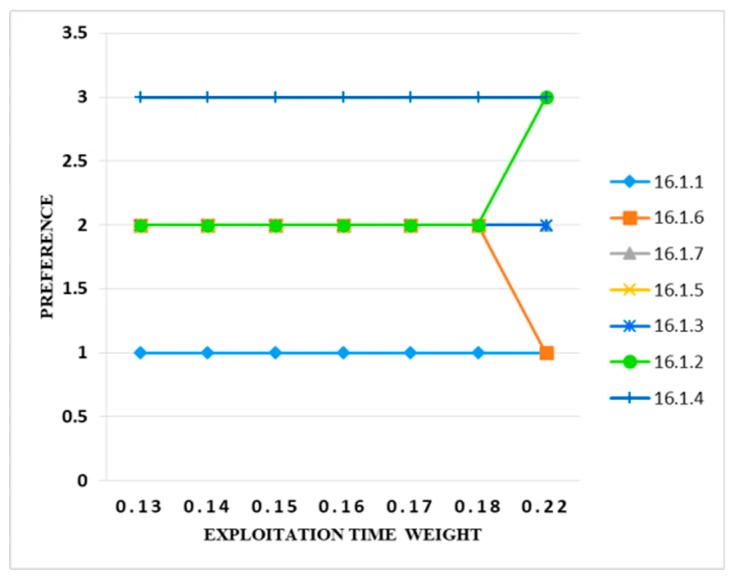
Sensitivity analysis of preference and exploitation time weight.

Exploitation Time also had a very minor impact on the results. The weight of the exploitation time criteria from 0.13 to 0.18 was varied. It was found that there was no change in the results when the criteria weight value was set to 0.22 which was 10%. Then the preference band of only two information security controls (i.e., 16.1.6 and 16.1.2) changed from 2 to 1 and 2 to 3, respectively. Hence, varying the weight of the exploitation time criteria also had a very minor effect. Figure 14 clearly depicts these results.

### 8.6. Maintenance Cost

**Figure 15:**
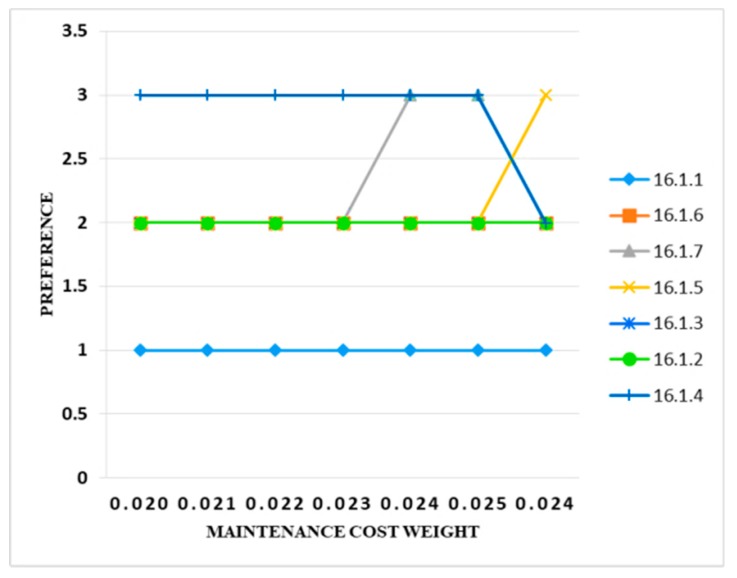
Sensitivity analysis of preference and maintenance cost weight.

Figure 15 shows that there was not much change in the results after varying the weights. The weight of the maintenance cost increased from 0.021 to 0.024, and it was observed that there was no change in the output. All the preference bands were intact. When the value increased to 0.024, only one information security control (i.e., 16.1.7) changed its preference band from 2 to 3, and when the weight was set to 0.025, then again there was no change in the output. In the resulting change to 10%, only three controls (i.e., 16.1.7, 16.1.4, and 16.1.5) changed their preference band. 

### 8.7. Maintenance Time

**Figure 16:**
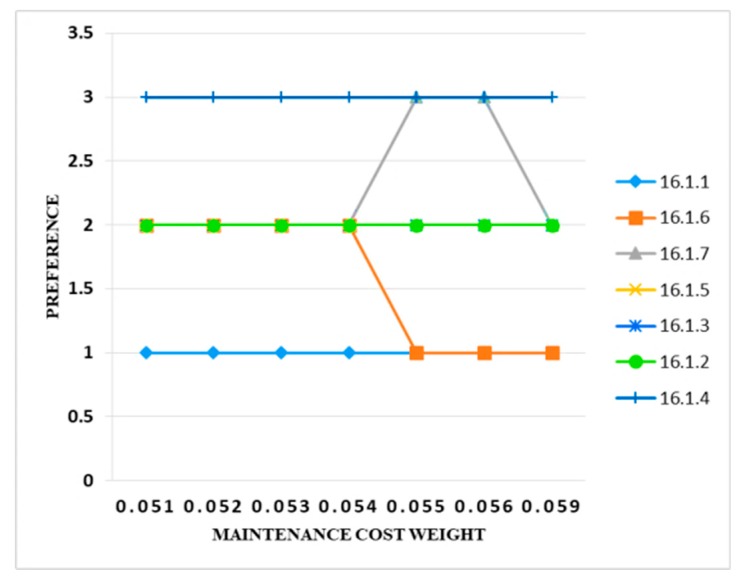
Sensitivity analysis of preference and maintenance cost weight.

The initial value of the maintenance time was 0.050. The value increased to 0.051, and there was no change. Similarly, when the value changed to 0.052 and 0.053, again there was no change in the preference band. When value increased to 0.054, only two controls (i.e., 16.1.6 and 16.1.7) changed their preference band from 2 to 1 and 2 to 3, respectively. When the weight value increased to 0.055 and 0.056, there was no change in the results, and when the criteria weight value finally increased to 0.059, only one preference band of the control (i.e., 16.1.7) changed from 3 to 2. Hence, the results show that the maintenance cost criteria had no uncertainty and were stable. Figure 16 clearly depicts the results. 

## 9. Comparison of Fuzzy AHP Technique with Other Existing Techniques 

During the literature review, the authors studied different stereotype criteria to compare different ISC prioritization techniques. The authors emphasized the comparison of MCDM techniques (AHP, ANP, TOPSIS, etc.) and overlooked the actual core and most relevant features. During the literature review, the author could not find any specific criteria in which ISCs selection techniques/methodologies were compared with others. The authors studied most cited information security control prioritization and selection techniques thoroughly and wrote their common and distinguished assessment points to formulate comprehensive criteria for the evaluations of ISCs methodologies. The authors critically analyzed each of the tasks covered in the relevant methodologies and collected the appropriate characteristics from other methodologies. After that, the authors combined them to formulate one task that covers all approaches opted by other methodologies. The said approach is very comprehensive, as the developed characteristics are taken from surveyed ISC methodologies.

The authors developed simple criteria to quantify each renowned information security framework, and the same was implemented on the adopted methodology in this paper to show its better effectiveness. The comparison of the existing ISC selection/prioritization methodologies with the presented methodology is given in Table 23. 

Table 23 shows the different methods used by the different authors to compare and prioritize ISCs and select the best one amongst a large number of ISCs. After comparing the abovementioned techniques, it was proved that the fuzzy AHP technique is most appropriate to prioritize/rank the ISCs, as it confronts ambiguity. It allows criteria, sub-criteria, pairwise comparison, normalization, weighting, and validation of the results through the consistency index and consistency ratio. The weighting and validation of each alternative maintains the hierarchy process and the massive number of criteria.

## 10. Conclusions

Accurate selection of ISCs and evaluation can help organizations in risk assessment exercises for their cloud networks integrated with wireless sensor networks. This paper presented a model to prioritize the ISCs of ISO/IEC 27002:2013 based on the FAHP to improve the efficiency of organizations in risk management. The AHP was used to create pairwise comparisons of the opinions of experts against the evaluation criteria. Thereafter, the outcome of the said comparisons were put into a matrix with experts’ opinions and was reciprocal. For experts’ opinions and to maintain the symmetry of the response, 11 generic steps were optimized. The prioritization of the information security controls and selection of the best one depended on numerous factors associated with the organization itself such as the deployed information security system and the environment. When information security best practices have too many information security controls, organizations search for relevant solutions to implement appropriate, cost-effective, and efficient information security controls which may increase the reliability of the system. The proposed model was developed based on factors found during the literature review. This model was optimized by many information security experts for the selection of information security controls. The model consists of seven criteria for the assessment of information security controls and prioritization. Triangular fuzzy numbers were used and assigned to the opinions of decision makers. In this study, an extended analysis method in conjunction with the FAHP was used. An in-depth analysis was performed for fuzzy evaluations to explain the synthetic priority weights for each criterion. The data used in this study were gathered using multi-criteria decision making methods. The results of this application show that the proposed model worked well. The results of the ranking of ISO/IEC 27002:2013 were also presented in this paper. The proposed model may be used for the evaluation and prioritization of other information security best practices such as National Institute of Standards and Technology - Special Publication 800-53 Revision 4, ISO/IEC 27001:2017, and Control Objectives for Information and Related Technology (COBIT) 5. The authors’ rationale was to study existing MCDM methodologies, select the best one to evaluate and prioritize the information security controls of ISO/IEC 27002:2013, and to develop a model to further prioritize information security controls of other standards.

## Figures and Tables

**Table 1:** Related research for information security risk and control selection.

Authors	Approaches	Field of Research
Shameli-Sendi et al. [60]	Fuzzy, MCDM and TOPSIS	Information Security Risk Assessment
Iryna Yevseyeva et al. [61]	Multiple-Criteria Decision Analysis and Multi-Attribute Utility Theory	The Behavior of Information Security
Irfan Syamsuddin and Junseok Hwang [62]	MCDM and AHP	Security Policy Decision Making
Hossein gharaee and Mahsa Agha Moheidin [63]	MADM, AHP, TOPSIS, and Fuzzy	Ranking of Information Security Risks
Brožová et al. [64]	MCDM and Analytic Network Process	Risk Analysis and Decision Making
Alireza Shameli Sendi et al. [65]	Fuzzy logic	Risk Assessment
Dong-mei Zhao et al. [66]	Fuzzy logic	Risk Assessment of the Network Security
Alexander A. Ganin et al. [67]	MCDA	Cybersecurity Risk Assessment and Management
Zeynep Filiz Eren-Dogu and Can Cengiz Celikoglu [68]	MCDM and AHP	Information Security Risk Assessment
Zhang Xinlan et al. [69]	MCDM and AHP	Information Security Risk Assessment
Jun-Jie Lv et al. [10]	PROMETHEE	Evaluation of ISCs
A. Ejnioui at el. [70]	MCDM and Grey System Theory	Evaluation of ISCs
Bao-Chyuan Guan at el. [71]	MCDM and ANP	Evaluation of Information Security Risks
Pedro Miguel Almeida Carvalho Costa [72]	MCDA	Evaluation of Cloud Services
James Ngeru et al. [73]	MCDM and Delphi AHP	Selecting Cloud Deployment Model
Hind Bangui et al. [74]	MCDA	Mobile Cloud Offloading
Chandrashekar Jatoth et al. [75]	Grey TOPSIS, MCDM	Selection of Cloud Services
Supriya M et al. [76]	MCDM and Fuzzy	Cloud Service Provider Selection
Yazır et al. [77]	Dynamic MCDA and PROMETHEE	Resource Allocation in Computing Clouds
Annette J Ruby et al. [78]	MCDM, Simple Additive Weighting (SAW), AHP	Ranking of Cloud Renderfarm Services
Luiz H. Nunes et al. [79]	SAW, TOPSIS, and VIKOR	IoT Resource Discovery
Suvendu Chandan Nayak et al. [80]	MCDM	Task Scheduling in Cloud Computing
Angel R. Otero et al. [81]	MCDM, Boolean	ISCs Selection
Suhaidi Hassan and Norliza Katuk [11]	MCDM, TOPSIS	ISCs Assessment
Angel Rafael Otero [82]	Fuzzy, MCDM, Grey Relation Analysis (GRA)	ISCs Assessment
Luís Almeida and Ana Respício [83]	MCDM, TOPSIS	Selection of ISCs
Artur Kierzkowski and Tomasz Kisiel [84]	Fuzzy, MCDM	Evaluation of ISCs
John Waxler [85]	MCDM, TOPSIS	Prioritizing of ISCs
Farzaneh Sadat Jalayer and Akbar Nabiollahi [86]	Fuzzy, MCDM, TOPSIS	Ranking of ISCs
Hamid Khajouei et al. [87]	Fuzzy, MCDM, AHP	Ranking of ISCs
Deepa Mani, et al. [88]	MCDM, AHP, Grey Relation Analysis	Selection of ISCs
Iryana et al. [89]	MDCM, TOPSIS	Two-Stage ISC Selection

**Table 2:** Fuzzy triangular linguistic scale and fuzzy numbers.

Scale (0–11)	Qualitative Evaluation	Triangular Fuzzy Scale	Reciprocal Scale
1	Equally Important	(1,1,1)	(1,1,1)
3	Moderate Important	(1,3,5)	(1/5, 1/3, 1)
5	Strongly Important	(3,5,7)	(1/7, 1/5, 1/3)
7	Very Strongly Important	(5,7,9)	(1/9, 1/7, 1/5)
9	Extremely Important	(7,9,11)	(1/11, 1/9, 1/7)

**Table 3:** Fuzzy evaluation matrix.

Criteria	C1	C2	C3	C4	C5	C6	C7
C1	(1,1,1)	(1/3,1/3,1/3)	(1,1,1)	(1,1,1)	(1,1,1)	(1,1,1)	(1,1,1)
C2	(3,3,3)	(1,1,1)	(1,3,5)	(1,3,5)	(1,3,5)	(3,5,7)	(1,3,5)
C3	(1,1,1)	(1/5,1/3,1)	(1,1,1)	(1,3,5)	(1,3,5)	(1,3,5)	(1,3,5)
C4	(1,1,1)	(1/5,1/3,1)	(1/5,1/3,1)	(1,1,1)	(1,1,1)	(3,5,7)	(1,3,5)
C5	(1,1,1)	(1/5,1/3,1)	(1/5,1/3,1)	(1,1,1)	(1,1,1)	(1,3,5)	(1,1,1)
C6	(1,1,1)	(1/7,1/5,1/3)	(1/5,1/3,1)	(1/7,1/5,1/3)	1/5,1/3,1	(1,1,1)	(1,1,1)
C7	(1,1,1)	(1,1/3,1)	(1/5,1/3,1)	(1/5,1/3,1)	(1,1,1)	(1,1,1)	(1,1,1)

**Table 4:** Final weights of each criteria.

(C_1_)	(C_2_)	(C_3_)	(C_4_)	(C_5_)	(C_6_)	(C_7_)
0.042	0.304	0.247	0.205	0.128	0.021	0.053

**Table 5:** Evaluation with respect to implementation time.

Alternatives	A1	A2	A3	A4
A1	(1, 1, 1)	(1, 3, 5)	(3, 5, 7)	(5, 7, 9)
A2	(1/5, 1/3, 1)	(1, 1, 1)	(1, 3, 5)	(3, 5, 7)
A3	(1/7, 1/5, 1/3)	(1/5, 1/3, 1)	(1, 1, 1)	(1, 3, 5)
A4	(1/9, 1/7, 1/5)	(1/7, 1/5, 1/3)	(1/5, 1/3, 1)	(1, 1, 1)

**Table 6:** Geometric means of alternatives in respect of implementation time.

Alternative	$r_{i}$		
A1	1.97	3.20	4.21
A2	0.88	1.50	2.43
A3	0.41	0.67	1.14
A4	0.24	0.31	0.51
Total	3.50	5.68	8.29
Reverse	0.29	0.18	0.12
Increasing order	0.12	0.18	0.29

**Table 7:** Relative fuzzy weights of the alternatives.

Alternative	$w_{i}$		
A1	0.237	0.564	1.205
A2	0.106	0.263	0.696
A3	0.050	0.118	0.325
A4	0.029	0.055	0.145

**Table 8:** Averaged and normalized values of the relative weights of the alternatives.

Alternative	$M_{i}$	$N_{i}$
A1	0.669	0.529
A2	0.355	0.281
A3	0.164	0.130
A4	0.076	0.060

**Table 9:** Evaluation with respect to effectiveness.

Alternatives	A1	A2	A3	A4
A1	(1, 1, 1)	(1, 3, 5)	(1, 1, 1)	(7, 9, 11)
A2	(1/5, 1/3, 1)	(1, 1, 1)	(1, 3, 5)	(3, 5, 7)
A3	(1, 1, 1)	(1/5, 1/3, 1)	(1, 1, 1)	(1, 3, 5)
A4	(1/11, 1/9, 1/7)	(1/7, 1/5, 1/3)	(1/5, 1/3, 1)	(1, 1, 1)

**Table 10:** Results of $r_{i},\;W_{i},\;M_{i}\;\mathrm{and}\;N_{i}$ in respect to the effectiveness criteria.

Alternative	$r_{i}$			$w_{i}$			$M_{i}$	$N_{i}$
A1	1.63	2.28	2.72	0.229	0.450	0.801	0.493	0.414
A2	0.88	1.50	2.43	0.124	0.295	0.715	0.378	0.318
A3	0.67	1.00	1.50	0.094	0.197	0.440	0.244	0.205
A4	0.23	0.29	0.47	0.032	0.058	0.137	0.076	0.064
Total	3.40	5.07	7.12					
Reverse	0.29	0.20	0.14					
Increasing order	0.14	0.20	0.29					

**Table 11:** Evaluation with respect to risk.

Alternatives	A1	A2	A3	A4
A1	(1, 1, 1)	(1, 3, 5)	(3, 5, 7)	(1, 3, 5)
A2	(1/5, 1/3, 1)	(1, 1, 1)	(1, 1, 1)	(3, 5, 7)
A3	(1/7, 1/5, 1/3)	(1, 1, 1)	(1, 1, 1)	(3, 5, 7)
A4	(1/5, 1/3, 1)	(1/7, 1/5, 1/3)	(1/7, 1/5, 1/3)	(1,1, 1)

**Table 12:** Results of $r_{i},\;W_{i},\;M_{i}\;\mathrm{and}\;N_{i}$ in respect to the risk criteria.

Alternative	$r_{i}$			$w_{i}$			$M_{i}$	$N_{i}$
A1	1.32	2.59	3.64	0.186	0.511	1.116	0.605	0.499
A2	0.88	1.14	1.63	0.124	0.224	0.499	0.283	0.233
A3	0.81	1.00	1.24	0.114	0.197	0.379	0.230	0.190
A4	0.25	0.34	0.58	0.036	0.067	0.177	0.093	0.077
Total	3.26	5.07	7.08					
Reverse	0.31	0.20	0.14					
Increasing order	0.14	0.20	0.31					

**Table 13:** Evaluation with respect to the budgetary constraints.

Alternatives	A1	A2	A3	A4
A1	(1, 1, 1)	(1, 3, 5)	(7, 9, 11)	(1, 3, 5)
A2	(1/5, 1/3, 1)	(1, 1, 1)	(1, 3, 5)	(1, 3, 5)
A3	(1/11, 1/9, 1/7)	(1/5, 1/3, 1)	(1, 1, 1)	(1, 1, 1)
A4	(1/5, 1/3, 1)	(1/5, 1/3, 1)	(1, 1, 1)	(1, 1, 1)

**Table 14:** Results of $r_{i},\;W_{i},\;M_{i\;}\mathrm{and}\;N_{i}$ in respect to the budgetary constraints.

Alternative	$r_{i}$			$w_{i}$			$M_{i}$	$N_{i}$
A1	1.63	3.00	4.07	0.205	0.563	1.310	0.692	0.527
A2	0.67	1.32	2.24	0.084	0.247	0.719	0.350	0.267
A3	0.37	0.44	0.61	0.046	0.082	0.198	0.109	0.083
A4	0.45	0.58	1.00	0.056	0.108	0.322	0.162	0.123
Total	3.11	5.33	7.92					
Reverse	0.32	0.19	0.13					
Increasing order	0.13	0.19	0.32					

**Table 15:** Evaluation with respect to the exploitation time.

Alternatives	A1	A2	A3	A4
A1	(1, 1, 1)	(3, 5, 7)	(1, 3, 5)	(1, 1, 1)
A2	(1/7, 1/5, 1/3)	(1, 1, 1)	(1, 1, 1)	(1, 3, 5)
A3	(1/5, 1/3, 1)	(1, 1, 1)	(1, 1, 1)	(1, 1, 1)
A4	(1, 1, 1)	(1/5, 1/3, 1)	(1, 1, 1)	(1, 1, 1)

**Table 16:** Results of $r_{i},\;W_{i},\;M_{i\;}\mathrm{and}\;N_{i}$ in respect to the exploitation time.

Alternative	$r_{i}$			$w_{i}$			$M_{i}$	$N_{i}$
A1	1.32	1.97	2.43	0.236	0.451	0.744	0.477	0.435
A2	0.61	0.88	1.14	0.110	0.202	0.348	0.220	0.200
A3	0.67	0.76	1.00	0.120	0.174	0.306	0.200	0.182
A4	0.67	0.76	1.00	0.120	0.174	0.306	0.200	0.182
Total	3.27	4.37	5.57					
Reverse	0.31	0.23	0.18					
Increasing order	0.18	0.23	0.31					

**Table 17:** Evaluation with respect to the maintenance cost.

Alternatives	A1	A2	A3	A4
A1	(1, 1, 1)	(1, 3, 5)	(1, 1, 1)	(1, 1, 1)
A2	(1/5, 1/3, 1)	(1, 1, 1)	(1, 1, 1)	(1, 3, 5)
A3	(1, 1, 1)	(1, 1, 1)	(1, 1, 1)	(1, 1, 1)
A4	(1, 1, 1)	(1/5, 1/3, 1)	(1, 1, 1)	(1, 1, 1)

**Table 18:** Results of $r_{i},\;W_{i},\;M_{i}\;\mathrm{and}\;N_{i}$ in respect to the exploitation time.

Alternative	$r_{i}$			$w_{i}$			$M_{i}$	$N_{i}$
A1	1.00	1.32	1.50	0.200	0.323	0.448	0.324	0.307
A2	0.67	1.00	1.50	0.134	0.245	0.448	0.276	0.261
A3	1.00	1.00	1.00	0.200	0.245	0.300	0.248	0.236
A4	0.67	0.76	1.00	0.134	0.186	0.300	0.207	0.196
Total	3.34	4.08	4.99					
Reverse	0.30	0.25	0.20					
Increasing order	0.20	0.25	0.30					

**Table 19:** Evaluation with respect to the mitigation time.

Alternatives	A1	A2	A3	A4
A1	(1, 1, 1)	(1, 3, 5)	(1, 3, 5)	(1, 1, 1)
A2	(1/5, 1/3, 1)	(1, 1, 1)	(1, 3, 5)	(1, 3, 5)
A3	(1/5, 1/3, 1)	(1/5, 1/3, 1)	(1, 1, 1)	(1, 1, 1)
A4	(1, 1, 1)	(1/5, 1/3, 1)	(1, 1, 1)	(1, 1, 1)

**Table 20:** Results of $r_{i},\;W_{i},\;M_{i}\;\mathrm{and}\;N_{i}$ in respect to the mitigation time.

Alternative	$r_{i}$			$w_{i}$			$M_{i}$	$N_{i}$
A1	1.00	1.73	2.24	0.155	0.395	0.803	0.451	0.360
A2	0.67	1.32	2.24	0.103	0.300	0.803	0.402	0.321
A3	0.45	0.58	1.00	0.069	0.132	0.359	0.187	0.149
A4	0.67	0.76	1.00	0.103	0.173	0.359	0.212	0.169
Total	2.78	4.39	6.47					
Reverse	0.36	0.23	0.15					
Increasing order	0.15	0.23	0.36					

**Table 21:** Consolidated results of all the alternatives in the light of each criterion.

Alternatives/Criteria Weights	(C1)	(C2)	(C3)	(C4)	(C5)	(C6)	(C7)	Total
	0.042	0.304	0.247	0.205	0.128	0.021	0.053	
A1	0.529	0.414	0.499	0.527	0.435	0.307	0.360	0.461
A2	0.281	0.318	0.233	0.267	0.200	0.261	0.321	0.269
A3	0.130	0.205	0.190	0.083	0.182	0.236	0.149	0.168
A4	0.060	0.064	0.077	0.123	0.182	0.196	0.169	0.103

**Table 22:** Ranked ISO/IEC 27002:2013 information security controls.

Control #	Fuzzy AHP Score	Rank	Preference
5.1.2	0.632	Rank 1	Preference 1
5.1.1	0.368	Rank 2	Preference 2
6.1.4	0.272	Rank 1	Preference 1
6.1.1	0.243	Rank 2	Preference 1
6.1.2	0.183	Rank 3	Preference 2
6.1.5	0.176	Rank 4	Preference 2
6.1.3	0.126	Rank 5	Preference 2
6.2.2	0.62	Rank 1	Preference 1
6.2.1	0.38	Rank 2	Preference 2
7.1.1	0.65	Rank 1	Preference 1
7.1.2	0.35	Rank 2	Preference 2
7.2.3	0.482	Rank 1	Preference 1
7.2.2	0.305	Rank 2	Preference 2
7.2.1	0.212	Rank 3	Preference 3
7.2.4	1.00	Rank 1	Preference 1
8.1.2	0.373	Rank 1	Preference 1
8.1.1	0.322	Rank 2	Preference 1
8.1.3	0.305	Rank 3	Preference 1
8.2.2	0.484	Rank 1	Preference 1
8.2.3	0.421	Rank 2	Preference 1
8.2.1	0.095	Rank 3	Preference 2
8.3.2	0.338	Rank 1	Preference 1
8.3.3	0.332	Rank 2	Preference 1
8.3.1	0.330	Rank 3	Preference 1
9.1.1	0.73	Rank 1	Preference 1
9.1.2	0.27	Rank 2	Preference 2
9.2.2	0.322	Rank 1	Preference 1
9.2.3	0.158	Rank 2	Preference 2
9.2.1	0.157	Rank 3	Preference 2
9.2.6	0.146	Rank 4	Preference 2
9.2.5	0.111	Rank 5	Preference 2
9.2.4	0.107	Rank 6	Preference 2
9.3.1	1.00	Rank 1	Preference 1
9.4.1	0.399	Rank 1	Preference 1
9.4.2	0.252	Rank 2	Preference 2
9.4.4	0.133	Rank 3	Preference 3
9.4.3	0.128	Rank 4	Preference 3
9.4.5	0.088	Rank 5	Preference 4
10.1.2	0.53	Rank 1	Preference 1
10.1.1	0.47	Rank 2	Preference 2
11.1.2	0.313	Rank 1	Preference 1
11.1.1	0.165	Rank 2	Preference 2
11.1.3	0.161	Rank 3	Preference 2
11.1.6	0.149	Rank 4	Preference 2
11.1.5	0.113	Rank 5	Preference 2
11.2.1	0.199	Rank 1	Preference 1
11.2.6	0.129	Rank 2	Preference 1
11.2.7	0.114	Rank 3	Preference 1
11.2.8	0.099	Rank 4	Preference 2
11.2.3	0.092	Rank 5	Preference 2
11.2.2	0.090	Rank 6	Preference 2
11.2.5	0.083	Rank 7	Preference 2
11.2.10	0.068	Rank 8	Preference 2
11.2.4	0.068	Rank 9	Preference 2
11.2.9	0.059	Rank 10	Preference 2
12.1.4	0.332	Rank 1	Preference 1
12.1.1	0.278	Rank 2	Preference 2
12.1.2	0.210	Rank 3	Preference 2
12.1.3	0.180	Rank 4	Preference 3
12.2.1	1.00	Rank 1	Preference 1
12.3.1	1.00	Rank 1	Preference 1
12.4.1	0.372	Rank 1	Preference 1
12.4.4	0.278	Rank 2	Preference 2
12.4.2	0.197	Rank 3	Preference 3
12.4.3	0.153	Rank 4	Preference 3
12.5.1	1.00	Rank 1	Preference 1
12.6.1	0.74	Rank 1	Preference 1
12.6.2	0.26	Rank 2	Preference 2
12.7.1	1.00	Rank 1	Preference 1
13.1.3	0.397	Rank 1	Preference 1
13.1.2	0.302	Rank 2	Preference 1
13.1.1	0.300	Rank 3	Preference 1
13.2.1	0.465	Rank 1	Preference 1
13.2.4	0.201	Rank 2	Preference 2
13.2.2	0.184	Rank 3	Preference 3
13.2.3	0.151	Rank 4	Preference 3
14.1.3	0.438	Rank 1	Preference 1
14.1.2	0.305	Rank 2	Preference 2
14.1.1	0.256	Rank 3	Preference 3
14.2.1	0.216	Rank 1	Preference 1
14.2.6	0.143	Rank 2	Preference 2
11.2.7	0.121	Rank 3	Preference 2
11.2.8	0.104	Rank 4	Preference 2
14.2.2	0.095	Rank 5	Preference 3
14.2.3	0.095	Rank 6	Preference 3
14.2.5	0.088	Rank 7	Preference 3
14.2.4	0.070	Rank 8	Preference 3
11.2.9	0.066	Rank 9	Preference 3
14.3.1	1.00	Rank 1	Preference 1
15.1.1	0.356	Rank 1	Preference 1
15.1.2	0.328	Rank 2	Preference 1
15.1.3	0.316	Rank 3	Preference 1
15.2.1	0.690	Rank 1	Preference 1
15.2.2	0.310	Rank 2	Preference 2
16.1.1	0.254	Rank 1	Preference 1
16.1.6	0.188	Rank 2	Preference 2
16.1.7	0.150	Rank 3	Preference 2
16.1.5	0.109	Rank 4	Preference 2
16.1.3	0.109	Rank 5	Preference 2
16.1.2	0.109	Rank 6	Preference 2
16.1.4	0.081	Rank 7	Preference 3
17.1.3	0.419	Rank 1	Preference 1
17.1.2	0.336	Rank 2	Preference 2
17.1.1	0.245	Rank 3	Preference 3
17.2.1	1.00	Rank 1	Preference 1
18.1.1	0.410	Rank 1	Preference 1
18.1.2	0.242	Rank 2	Preference 2
18.1.4	0.131	Rank 3	Preference 3
18.1.3	0.131	Rank 4	Preference 3
18.1.5	0.086	Rank 5	Preference 4
18.2.3	0.419	Rank 1	Preference 1
18.2.2	0.336	Rank 2	Preference 2
18.2.1	0.245	Rank 3	Preference 3

**Table 23:** Comparison of fuzzy AHP with other methodologies for ISCs selection/prioritization.

Criteria	[40]	[100]	[10]	[11]	[101]	[83]	Fuzzy AHP Technique of this Article
Fuzzy Logic	No	Yes	No	No	No	No	Yes
Pairwise Comparison	No	No	No	No	No	No	Yes
Goals of the Study	No	No	Yes	Yes	No	Yes	Yes
Weighting of Criteria	No	No	No	Yes	No	No	Yes
Validation of Results	No	No	No	Yes	Yes	No	Yes
Systematic	Yes	Yes	Yes	Yes	Yes	Yes	Yes
Complexity	Moderate	Moderate	Low	Moderate	Low	Low	High
Elasticity	Yes	Yes	Yes	Yes	No	Yes	Yes
Consistency	No	No	Yes	No	No	Yes	Yes
Homogeneity	Yes	No	Yes	No	Yes	No	Yes
Independence	No	Yes	Yes	Yes	Yes	Yes	Yes
Computational Requirement	High	High	Low	Moderate	Low	High	High
Probability and Possibility	No	No	Yes	No	No	Yes	Yes

## References

[1] Ahmed K., Gregory M. Integrating Wireless Sensor Networks with Cloud Computing. Proceedings of the 2011 Seventh International Conference on Mobile Ad-hoc and Sensor Networks.

[2] Arroyo P., Herrero J.L., Suárez J.I., Lozano J. (2019). Wireless Sensor Network Combined with Cloud Computing for Air Quality Monitoring. Sensors.

[3] Swathi B.S., Guruprasad H. (2014). Integration of wireless sensor networks and cloud computing. Int. J. Comput. Sci..

[4] Mhatre L., Rai N. Integration between wireless sensor and cloud. Proceedings of the 2017 International Conference on I-SMAC (IoT in Social, Mobile, Analytics and Cloud) (I-SMAC).

[5] Jassas M. (2015). A Framework for Integrating Wireless Sensors and Cloud Computing. Ph.D. Thesis.

[6] Alharbe N., Atkins A.S., Champion J. Use of cloud computing with wireless sensor networks in an Internet of Things environment for a smart hospital network. Proceedings of the Seventh International Conference on eHealth, Telemedicine, and Social Medicine.

[7] Srivastava R., Hasan M. (2018). Comparative study on Integration of Wireless Sensor Network With Cloud Computing. Int. J. Adv. Res. Comput. Sci..

[8] Shah S.H., Khan F.K., Ali W., Khan J., Hussain S.S., Kabeer K.F., Wajid A., Jamshed K. A new framework to integrate wireless sensor networks with cloud computing. Proceedings of the 2013 IEEE Aerospace Conference.

[9] Tariq M.I., Tayyaba S., Rasheed H., Ashraf M.W. (2017). Factors influencing the Cloud Computing adoption in Higher Education Institutions of Punjab, Pakistan.

[10] Tariq M.I., Tayyaba S., Hashmi M.U., Ashraf M.W., Mian N.A. (2017). Agent Based Information Security Threat Management Framework for Hybrid Cloud Computing. IJCSNS.

[11] Tariq M.I., Tayyaba S., Ashraf M.W., Rasheed H. (2017). Risk Based NIST Effectiveness Analysis for Cloud Security. Bahria Univ. J. Inf. Commun. Technol. (BUJICT).

[12] Tariq M.I. (2012). Towards information security metrics framework for cloud computing. Int. J. Cloud Comput. Serv. Sci..

[13] Tariq M.I. (2018). Analysis of the Effectiveness of Cloud Control Matrix for Hybrid Cloud Computing. Int. J. Future Gener. Commun. Netw..

[14] Tariq M.I. (2019). Agent Based Information Security Framework for Hybrid Cloud Computing. KSII Trans. Internet Inf. Syst..

[15] Llanso T., McNeil M., Noteboom C. Multi-Criteria Selection of Capability-Based Cybersecurity Solutions. Proceedings of the 52nd Hawaii International Conference on System Sciences.

[16] Bharathi S.V. (2017). Prioritizing and ranking the big data information security risk spectrum. Glob. J. Flex. Syst. Manag..

[17] Patil A.N. (2018). FUZZY AHP Methodology and its sole applications. Int. J. Manag. Res. Rev..

[18] Chen J.-F., Hsieh H.-N., Do Q.H. (2015). Evaluating teaching performance based on fuzzy AHP and comprehensive evaluation approach. Appl. Soft Comput..

[19] Butt S.A., Tariq M.I., Jamal T., Ali A., Martinez J.L.D., De-La-Hoz-Franco E. (2019). Predictive Variables for Agile Development Merging Cloud Computing Services. IEEE Access.

[20] Tariq M.I., Haq D., Iqbal J. (2013). SLA Based Information Security Metric for Cloud Computing from COBIT 4.1 Framework. Int. J. Comput. Netw. Commun. Secur..

[21] Chen S.-J., Hwang C.-L. (1992). Fuzzy Multiple Attribute Decision Making. Lecture Notes in Economics and Mathematical Systems.

[22] Ruiz-Padillo A., Pasqual F.M., Uriarte A.M.L., Cybis H.B.B. (2018). Application of multi-criteria decision analysis methods for assessing walkability: A case study in Porto Alegre, Brazil. Transp. Res. Part D Transp. Environ..

[23] Saaty T.L. (1988). What is the analytic hierarchy process?. Mathematical Models for Decision Support.

[24] Kubler S., Robert J., Derigent W., Voisin A., Le Traon Y. (2016). A state-of the-art survey & testbed of fuzzy AHP (FAHP) applications. Expert Syst. Appl..

[25] Calabrese A., Costa R., Levialdi N., Menichini T. (2019). Integrating sustainability into strategic decision-making: A fuzzy AHP method for the selection of relevant sustainability issues. Technol. Forecast. Soc. Chang..

[26] Wang H., Jiang Z., Zhang H., Wang Y., Yang Y., Li Y. (2019). An integrated MCDM approach considering demands-matching for reverse logistics. J. Clean. Prod..

[27] Perumal B., Rajasekaran P., Ramalingam H. WSN integrated cloud for automated telemedicine (ATM) based e-healthcare applications. Proceedings of the 4th International Conference on Bioinformatics and Biomedical Technology (IPCBEE’12).

[28] Alcaraz C., Najera P., Lopez J., Roman R. Wireless sensor networks and the internet of things: Do we need a complete integration?. Proceedings of the 1st International Workshop on the Security of the Internet of Things (SecIoT’10).

[29] Othman M.F., Shazali K. (2012). Wireless Sensor Network Applications: A Study in Environment Monitoring System. Procedia Eng..

[30] Piyare R., Lee S.-R. (2013). Towards Internet of Things (IOTS): Integration of Wireless Sensor Network to Cloud Services for Data Collection and Sharing. Int. J. Comput. Netw. Commun..

[31] Agrawal A., Kaushal S. A Study on Integration of Wireless Sensor Network and Cloud Computing: Requirements, Challenges and Solutions. Proceedings of the Sixth International Conference on Computer and Communication Technology.

[32] Piyare R., Park S., Maeng S.Y., Park S.H., Oh S.C., Gil Choi S., Choi H.S., Lee S.R. Integrating Wireless Sensor Network into Cloud services for real-time data collection. Proceedings of the 2013 International Conference on ICT Convergence (ICTC).

[33] Al-Amri A., Ansari W.S., Hassan M.M., Hossain M.S., Alelaiwi A., Hossain M.A. (2013). A Survey on Sensor-Cloud: Architecture, Applications, and Approaches. Int. J. Distrib. Sens. Netw..

[34] Hassan M.M., Song B., Huh E.-N. A framework of sensor-cloud integration opportunities and challenges. Proceedings of the 3rd international conference on Mobile and ubiquitous multimedia-MUM ’04; Association for Computing Machinery (ACM).

[35] Manea G., Popa S. (2016). Integration of sensor networks in cloud computing. UPB Sci. Bull. Ser. C.

[36] Khoufi I., Mahfoudh S., Minet P., Laouiti A. (2015). Data gathering architecture for temporary worksites based on a uniform deployment of wireless sensors. Int. J. Sens. Networks.

[37] Kurschl W., Beer W. Combining cloud computing and wireless sensor networks. Proceedings of the 11th International Conference on Distributed Smart Cameras—ICDSC 2017, Association for Computing Machinery (ACM).

[38] Santhanam M.V., Shanmugam M.D. (2018). Integrating Wireless Sensor Networks with Cloud Computing and Emerging its platforms using Middleware Services. Int. Res. J. Eng. Technol..

[39] Ahmed K., Alexandrov V. (2011). Identity and Access Management in Cloud Computing. Guide to Security in SDN and NFV.

[40] Barnard L., Von Solms R. (2000). A Formalized Approach to the Effective Selection and Evaluation of Information Security Controls. Comput. Secur..

[41] Van Der Haar H., Solms R. (2003). A model for deriving information security control attribute profiles. Comput. Secur..

[42] Saint-Germain R. (2005). Information security management best practice based on ISO/IEC 17799. Inf. Manag. J. Prairie Village.

[43] Da Veiga A., Eloff J. (2007). An Information Security Governance Framework. Inf. Syst. Manag..

[44] Al-Safwani N., Fazea Y., Ibrahim H. (2018). ISCP: In-depth model for selecting critical security controls. Comput. Secur..

[45] Brauers W.K., Zavadskas E.K. (2006). The MOORA method and its application to privatization in a transition economy. Control Cybern..

[46] Saaty T.L. (1990). How to make a decision: The analytic hierarchy process. Eur. J. Oper. Res..

[47] Tzeng G.-H., Chiang C.-H., Li C.-W. (2007). Evaluating intertwined effects in e-learning programs: A novel hybrid MCDM model based on factor analysis and DEMATEL. Expert Syst. Appl..

[48] Gencer C., Gürpinar D. (2007). Analytic network process in supplier selection: A case study in an electronic firm. Appl. Math. Model..

[49] Hwang C.-L., Yoon K. (1981). Methods for Multiple Attribute Decision Making. Lecture Notes in Economics and Mathematical Systems.

[50] Riantaphyllou E. (2000). Multi-criteria decision making methods. Multi-Criteria Decision Making Methods: A Comparative Study.

[51] Garvey P.R. (2008). Analytical Methods for Risk Management: A Systems Engineering Perspective.

[52] Firouzabadi A.K., Ghazimatin E. (2013). Application of Preference Ranking Organization Method for Enrichment Evaluation Method in Energy Planning—Regional Level 16. Iran. J. Fuzzy Syst..

[53] Zavadskas E.K., Antuchevičienė J., Kapliński O. (2016). Multi-Criteria Decision Making in Civil Engineering: Part I– A State-of-the-Art Survey. Eng. Struct. Technol..

[54] Opricovic S., Tzeng G.-H. (2004). Compromise solution by MCDM methods: A comparative analysis of VIKOR and TOPSIS. Eur. J. Oper. Res..

[55] Opricovic S., Tzeng G.-H. (2007). Extended VIKOR method in comparison with outranking methods. Eur. J. Oper. Res..

[56] Sun C.-C. (2010). A performance evaluation model by integrating fuzzy AHP and fuzzy TOPSIS methods. Expert Syst. Appl..

[57] Chen C.-T. (2000). Extensions of the TOPSIS for group decision-making under fuzzy environment. Fuzzy Sets Syst..

[58] Tavana M., Zandi F., Katehakis M.N. (2013). A hybrid fuzzy group ANP–TOPSIS framework for assessment of e-government readiness from a CiRM perspective. Inf. Manag..

[59] Mir M.A., Ghazvinei P.T., Sulaiman N., Basri N., Saheri S., Mahmood N., Jahan A., Begum R., Aghamohammadi N. (2016). Application of TOPSIS and VIKOR improved versions in a multi criteria decision analysis to develop an optimized municipal solid waste management model. J. Environ. Manag..

[60] Shameli-Sendi A. (2012). Fuzzy Multi-Criteria Decision-Making for Information Security Risk Assessment. Open Cybern. Syst. J..

[61] Yevseyeva I., Morisset C., Groß T., Van Moorsel A. (2014). A Decision Making Model of Influencing Behavior in Information Security. Formal Aspects of Component Software.

[62] Syamsuddin I., Hwang J. (2010). The Use of AHP in Security Policy Decision Making: An Open Office Calc Application. J. Softw..

[63] Gharaee H., Agha M.M. (2015). Designing of Multi Criteria Decision Making Model for Improve Ranking of Information Security Risks. Signal Data Process..

[64] Brožová H., Dse F., Šup L., Rydval J., Sadok M., Bednar P. (2016). Information Security Management: ANP Based Approach for Risk Analysis and Decision Making. Agris Line Pap. Econ. Inform..

[65] Sendi A.S., Jabbarifar M., Shajari M., Dagenais M. FEMRA: Fuzzy Expert Model for Risk Assessment. Proceedings of the 2010 Fifth International Conference on Internet Monitoring and Protection.

[66] Zhao D.M., Wang J.H., Ma J.F. Fuzzy Risk Assessment of the Network Security. Proceedings of the 2006 International Conference on Machine Learning and Cybernetics.

[67] Ganin A.A., Quach P., Panwar M., Collier Z.A., Keisler J.M., Marchese D., Linkov I. (2017). Multicriteria Decision Framework for Cybersecurity Risk Assessment and Management. Risk Anal..

[68] Eren-Dogu Z.F., Celikoglu C.C. (2012). Information Security Risk Assessment: Bayesian Prioritization for AHP Group Decision Making. Int. J. Innov. Comput. Inf. Control.

[69] Xinlan Z., Zhifang H., Guangfu W., Xin Z. Information Security Risk Assessment Methodology Research: Group Decision Making and Analytic Hierarchy Process. Proceedings of the 2010 Second world congress on software engineering.

[70] Ejnioui A., Otero A.R., Tejay G., Otero C., Qureshi A. A Multi-attribute Evaluation of Information Security Controls in Organizations Using Grey Systems Theory. Proceedings of the Steering Committee of The World Congress in Computer Science, Computer Engineering and Applied Computing (WorldComp).

[71] Guan B.-C., Lo C.-C., Wang P., Hwang J.-S. Evaluation of information security related risks of an organization—The application of the multi-criteria decision-making method. Proceedings of the IEEE 37th Annual 2003 International Carnahan Conference onSecurity Technology.

[72] Krishankumar R., Ravichandran K.S., Tyagi S.K. (2020). Solving cloud vendor selection problem using intuitionistic fuzzy decision framework. Neural Computing and Applications.

[73] Ngeru J., Bardhan T.K. (2015). Selecting Cloud Deployment Model Using a Delphi Analytic Hierarchy Process (DAHP). Ind. Syst. Eng. Rev..

[74] Bangui H., Ge M., Buhnova B., Rakrak S., Raghay S., Pitner T. (2017). Multi-Criteria Decision Analysis Methods in the Mobile Cloud Offloading Paradigm. J. Sens. Actuator Netw..

[75] Jatoth C., Gangadharan G.R., Fiore U., Buyya R. (2018). SELCLOUD: A hybrid multi-criteria decision-making model for selection of cloud services. Soft Comput..

[76] Supriya M., Sangeeta K., Patra G. (2016). Trustworthy Cloud Service Provider Selection using Multi Criteria Decision Making Methods. Eng. Lett..

[77] Yazir Y.O., Matthews C., Farahbod R., Neville S., Guitouni A., Ganti S., Coady Y., Yazır Y.O. Dynamic Resource Allocation in Computing Clouds Using Distributed Multiple Criteria Decision Analysis. Proceedings of the 2010 IEEE 3rd International Conference on Cloud Computing.

[78] Annette J.R., Banu A., Chandran P.S. (2016). Comparison of Multi Criteria Decision Making Algorithms for Ranking Cloud Renderfarm Services. Indian J. Sci. Technol..

[79] Nunes L.H., Estrella J.C., Perera C., Delbem A.C.B., Reiff-Marganiec S. (2016). Multi-criteria IoT resource discovery: A comparative analysis. Softw. Pr. Exp..

[80] Nayak S.C., Parida S., Tripathy C., Pattnaik P.K. (2018). Task Scheduling Mechanism Using Multi-criteria Decision-making Technique, MACBETH in Cloud Computing. Advances in Intelligent Systems and Computing.

[81] Otero A., Otero C., Qureshi A. (2010). A Multi-Criteria Evaluation of Information Security Controls Using Boolean Features. Int. J. Netw. Secur. Its Appl..

[82] Otero A. (2015). An information security control assessment methodology for organizations’ financial information. Int. J. Account. Inf. Syst..

[83] Almeida L., Respicio A. (2018). Decision support for selecting information security controls. J. Decis. Syst..

[84] Kierzkowski A., Kisiel T. (2017). Evaluation of a Security Control Lane with the Application of Fuzzy Logic. Procedia Eng..

[85] Waxler J. (2018). Prioritizing Security Controls Using Multiple Criteria Decision Making for Home Users. Ph.D Thesis.

[86] Jalayer F.S., Nabiollahi A. (2016). Ranking Criteria of Enterprise Information Security Architecture Using Fuzzy Topsis. Int. J. Comput. Sci. Inf. Technol..

[87] Khajouei H., Kazemi M., Moosavirad S.H. (2016). Ranking information security controls by using fuzzy analytic hierarchy process. Inf. Syst. e-Business Manag..

[88] Choo K.K., Mubarak S., Mani D. Selection of information security controls based on AHP and GRA. Proceedings of the 18th Pacific Asia Conference on Information Systems.

[89] Yevseyeva I., Basto-Fernandes V., Van Moorsel A., Janicke H., Emmerich M. (2016). Two-stage Security Controls Selection. Procedia Comput. Sci..

[90] Leśniak A., Kubek D., Plebankiewicz E., Zima K., Belniak S. (2018). Fuzzy AHP Application for Supporting Contractors’ Bidding Decision. Symmetry.

[91] Zhu K.-J., Jing Y., Chang D.-Y. (1999). A discussion on Extent Analysis Method and applications of fuzzy AHP. Eur. J. Oper. Res..

[92] Chou Y.-C., Yen H.-Y., Dang V.T., Sun C.-C. (2019). Assessing the Human Resource in Science and Technology for Asian Countries: Application of Fuzzy AHP and Fuzzy TOPSIS. Symmetry.

[93] Diouf M., Kwak C. (2018). Fuzzy AHP, DEA, and Managerial Analysis for Supplier Selection and Development; From the Perspective of Open Innovation. Sustainability.

[94] Saha O., Chakraborty A., Banerjee J.S. (2018). A Fuzzy AHP Approach to IT-Based Stream Selection for Admission in Technical Institutions in India. Advances in Intelligent Systems and Computing.

[95] Aslantaş S., Tepe S., Mertoğlu B. (2019). A Fuzzy Based Risk Assessment Model with a Real Case Study.

[96] Fu S., Zhou H. The information security risk assessment based on AHP and fuzzy comprehensive evaluation. Proceedings of the 2011 IEEE 3rd International Conference on Communication Software and Networks.

[97] Wang J.-S., Fu Y., Wu X.-P. (2011). Research on security risk assessment of information system based on improved fuzzy AHP. Huoli yu Zhihui Kongzhi.

[98] Zhao D.-M., Wang J.-H., Wu J., Ma J.-F. Using Fuzzy Logic and Entropy Theory to Risk Assessment of the Information Security. Proceedings of the 2005 International Conference on Machine Learning and Cybernetics.

[99] Wang J.-S., Liu C.-H., Lin G.T. How to manage information security in cloud computing. Proceedings of the 2011 IEEE Conference on Systems, Man, and Cybernetics.

[100] Amine M., El Manouar A., Marhraoui M.A. (2017). An AHP Model towards an Agile Enterprise. Int. J. Adv. Comput. Sci. Appl..

[101] Otero A., Tejay G., Otero L.D., Ruiz-Torres A. A fuzzy logic-based information security control assessment for organizations. Proceedings of the 2012 IEEE Conference on Open Systems.

